# Modeling SARS-CoV-2 and influenza infections and antiviral treatments in human lung epithelial tissue equivalents

**DOI:** 10.1038/s42003-022-03753-7

**Published:** 2022-08-12

**Authors:** Hoda Zarkoob, Anna Allué-Guardia, Yu-Chi Chen, Andreu Garcia-Vilanova, Olive Jung, Steven Coon, Min Jae Song, Jun-Gyu Park, Fatai Oladunni, Jesse Miller, Yen-Ting Tung, Ivan Kosik, David Schultz, James Iben, Tianwei Li, Jiaqi Fu, Forbes D. Porter, Jonathan Yewdell, Luis Martinez-Sobrido, Sara Cherry, Jordi B. Torrelles, Marc Ferrer, Emily M. Lee

**Affiliations:** 1 3D Tissue Bioprinting Lab, Division of Preclinical Innovation, National Center for Advancing Translational Sciences, National Institutes of Health, Rockville, MD USA; 2 Host-Pathogen Interactions and Population Health Programs, Texas Biomedical Research Institute, San Antonio, TX USA; 3 Biomedical Ultrasonics & Biotherapy Laboratory, Institute of Biomedical Engineering, Department of Engineering Science, University of Oxford, Headington, UK; 4 Molecular Genomics Core, National Institute of Child Health and Human Development, National Institutes of Health, Rockville, MD USA; 5 Department of Biochemistry and Biophysics, University of Pennsylvania, Philadelphia, PA USA; 6 Department of Pathology and Laboratory Medicine, University of Pennsylvania, Philadelphia, PA USA; 7 Department of Microbiology, University of Pennsylvania, Philadelphia, PA USA; 8 National Institute for Allergies and Infectious Diseases, National Institutes of Health, Bethesda, MD USA; 9 High Throughput Screening Core, University of Pennsylvania, Philadelphia, PA USA; 10 Section on Molecular Dysmorphology, Division of Translational Medicine, Eunice Kennedy Shriver National Institute of Child Health and Human Development, National Institutes of Health, Department of Health and Human Services, Bethesda, MD 20892 USA

**Keywords:** SARS-CoV-2, High-throughput screening

## Abstract

There is a critical need for physiologically relevant, robust, and ready-to-use in vitro cellular assay platforms to rapidly model the infectivity of emerging viruses and develop new antiviral treatments. Here we describe the cellular complexity of human alveolar and tracheobronchial air liquid interface (ALI) tissue models during SARS-CoV-2 and influenza A virus (IAV) infections. Our results showed that both SARS-CoV-2 and IAV effectively infect these ALI tissues, with SARS-CoV-2 exhibiting a slower replication peaking at later time-points compared to IAV. We detected tissue-specific chemokine and cytokine storms in response to viral infection, including well-defined biomarkers in severe SARS-CoV-2 and IAV infections such as CXCL10, IL-6, and IL-10. Our single-cell RNA sequencing analysis showed similar findings to that found in vivo for SARS-CoV-2 infection, including dampened IFN response, increased chemokine induction, and inhibition of MHC Class I presentation not observed for IAV infected tissues. Finally, we demonstrate the pharmacological validity of these ALI tissue models as antiviral drug screening assay platforms, with the potential to be easily adapted to include other cell types and increase the throughput to test relevant pathogens.

## Abstract (web summary)

Human alveolar and tracheobronchial epithelial air liquid interface (ALI) tissues are used as models to examine cellular responses to SARS-CoV-2 and influenza A virus infections and as antiviral drug screening assay platforms.

## Introduction

Newly emerging viral pathogens such as severe acute respiratory syndrome coronavirus 2 (SARS-CoV-2) and other re-emerging respiratory viral threats, including influenza viruses, are a constant burden to human public health. Two years after the initial discovery of the novel SARS-CoV-2^1,2^, the coronavirus disease 2019 (COVID-19) pandemic remains a public health problem. While global vaccination efforts are being implemented, they are challenged by the emergence of new viral variants of concern (VoC) that can potentially escape immunity^3–6^. Due to immune escape as well as the potential for newly emergent SARS-CoV-2 VoC, and in spite of recent FDA drug approvals and emergency use authorizations for anti-SARS-CoV-2 drugs^7–9^, there remains a critical need to develop effective drug treatments^10^.

Several established cell lines permissive to SARS-CoV-2 infection in vitro are used for high-throughput antiviral drug screening (HTS), including the African green monkey kidney Vero E6 cells, human hepatoma Huh7 and Huh7.5 cells, colon carcinoma Caco2 cells, lung adenocarcinoma Calu-3 cells, human angiotensin converting enzyme 2 (hACE2) overexpressing adenocarcinoma A549 cells, HEK293T cells, and several other non-human cell lines^4,11–18^. While these cell lines are important tools for viral research, there is now evidence that many of the antiviral drug activities discovered are limited to the cells used for screening. For example, while hydroxychloroquine potently blocks SARS-CoV-2 infection of Vero E6 and Huh7 cells^4,19^, it is inactive in human Calu-3 lung cells^12,20^, and in both prophylactic and therapeutic treatment of SARS-CoV-2 in either rhesus macaque or golden Syrian hamster models^21–23^. Indeed, hydroxychloroquine is also ineffective in randomized COVID-19 human clinical trials^24,25^. Animal models have been developed for pre-clinical drug development of COVID-19, but the low-throughput of high biocontainment in vivo models limits their use for drug screening. There remains therefore a critical need for in vitro pre-clinical assays that are highly predictive of clinical drug efficacy, which can be used to prioritize compound selection for animal testing.

Air-liquid interface (ALI) lung tissues provide a bridge between cultured cell lines and animal models^26–32^. In addition to more closely replicating the physiological environment of the human lung epithelium, ALI tissues support the replication of human coronaviruses (HCoV) with limited host cell range, including HCoV-229E, HCoV-HKU1, HCoV-NL63, and HCoV-OC43^31,33–36^. To address the current shortcomings of traditional antiviral screening models, we investigated the use and predictive efficacy of commercially available ALI tissues modeling two regions of the lower respiratory tract – the tracheobronchial region and the alveolar region – in the context of SARS-CoV-2 and influenza A virus (IAV) infections. Here we demonstrate that both ALI tissues are susceptible to viral infection, with SARS-CoV-2 showing a slower replication rate compared to IAV, mimicking human infection. Further, using phenotypic and genotypic approaches we defined the top modulated pathways involved in induction of tissue-specific cytokine and chemokines in both ALI tissues in response to SARS-CoV-2 and IAV infections.

We further demonstrated the pharmacological validity of these ALI tissue models as in vitro antiviral drug screening platforms using viral protein immunostaining fluorescence imaging assays, viral RNA quantification, and live virus titration. Importantly, we showed that the in vitro ALI tissues faithfully recapitulated antiviral effects of remdesivir and the lack of antiviral effects of hydroxychloroquine, further supporting the use of ALI tissues for antiviral drug validations.

## Results

### Lung epithelial cell expression of known SARS-CoV-2 host entry co-factors in human tracheobronchial and alveolar ALI tissues

**Fig. 1:**
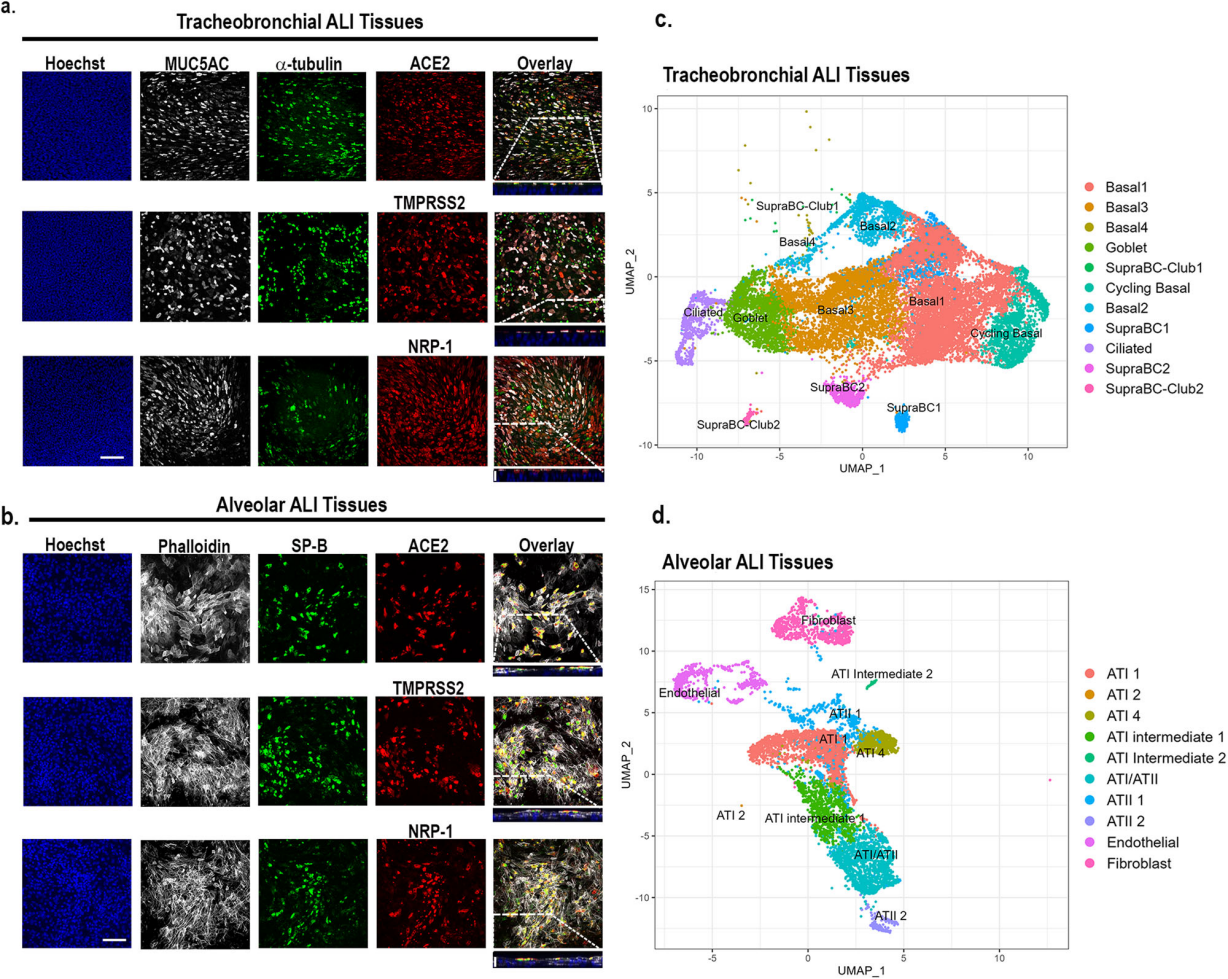
Apical expression patterns of known SARS-CoV-2 entry co-factors in tracheobronchial and alveolar ALI tissue equivalents. Post-day 21 tissues were stained with antibodies targeting ACE2, TMPRSS2, and NRP-1, as well as tissue-specific markers. **a** Representative stained images of tracheobronchial ALI tissues with Hoechst (nuclei marker, blue), MUC5AC (goblet cell marker, white) α -tubulin (ciliated cell marker, green), and ACE2 (top panel, red), TMPRSS2 (middle panel, red) or NRP-1 (bottom panel, red). The overlay image represents the maximum intensity projection of stained markers. The y/z plane cross section taken from the highlighted portion shows the selective apical expression of ACE2, TMPRSS2, and NRP-1. **b** Representative stained images of alveolar ALI tissues with Hoechst (nuclei marker, blue), phalloidin (f-actin, white), surfactant protein B (SP-B, ATII/pneumonocyte type II cell marker, green) and co-stained with ACE2 (top panel, red), TMPRSS2 (middle panel, red) or NRP-1 (bottom panel, red). The overlay image represents the maximum intensity projection of stained markers and a y/z plane cross section from the highlighted portion shows the selective apical expression of hACE2, TMPRSS2, or NRP-1 in the tissues, contrasted to phalloidin, which is present throughout the tissue cross-section. Scale bar is 100 μm. Cross-section scale bar is 20 μm. Uniform Manifold Approximation and Projection plots (UMAP plots) of scRNAseq data of dissociated (**c**) tracheobronchial or (**d**) alveolar ALI tissues.

We first assessed that the ALI tissues (which defines both tracheobronchial and alveolar ALI tissues in text) had differentiated lung epithelial cells by immunofluorescence and scRNAseq. The ALI tracheobronchial tissues are comprised of ciliated cells (α-tubulin+), goblet or secretory cells (MUC5AC^+^), and basal cells (KRT5^+^), as shown in Fig. 1a and c^37,38^. ALI alveolar tissues are comprised of lung epithelial alveolar type I (AQP5^+^, not shown) and type II cells (SP-B^+^), pulmonary fibroblasts, and endothelial cells (Fig. 1b, d). Next, we investigated the expression of putative SARS-CoV-2 and IAV cellular receptors^39–42^ in the ALI tissues by immunofluorescence (Supplementary Table 1). This included ACE2, a known host entry factor for both SARS-CoV and SARS-CoV-2^40,43^ highly expressed in alveolar type II cells (ATII) and also at lower levels in ciliated cells and goblet cells^37,38,44^, as well as Type II transmembrane serine protease TMPRSS2^40,45–47,48^, and transmembrane glycoprotein neuropilin-1 (NRP-1)^41,42^. ACE2 was expressed in the apical epithelium of both tracheobronchial (Fig. 1a, top row) and alveolar ALI tissues (Fig. 1b, top row). We also observed robust TMPRSS2 and NRP-1 expression in both tissues (Fig. 1a, b, second and third rows). In addition, we observed positive co-staining of ACE2, TMPRSS2, or NRP-1 with α-tubulin+ ciliated and MUC5AC + goblet cells in tracheobronchial ALI tissues (Fig. 1a), and SP-B^+^ cells in alveolar ALI tissues (Fig. 1b). A small fraction of cells that were SP-B negative also expressed SARS-CoV-2 entry cofactors. The scRNAseq analysis of dissociated tissue cultures confirmed the existence of these major cell types in these ALI tissues (Fig. 1c, d). Major cell type groupings, differentially expressed genes (DEGs) used for the cell type grouping, and number of cells identified per cell ID can be found in Supplementary Table 2.

### Human tracheobronchial and alveolar ALI tissues support productive SARS-CoV-2 or IAV infection

**Fig. 2:**
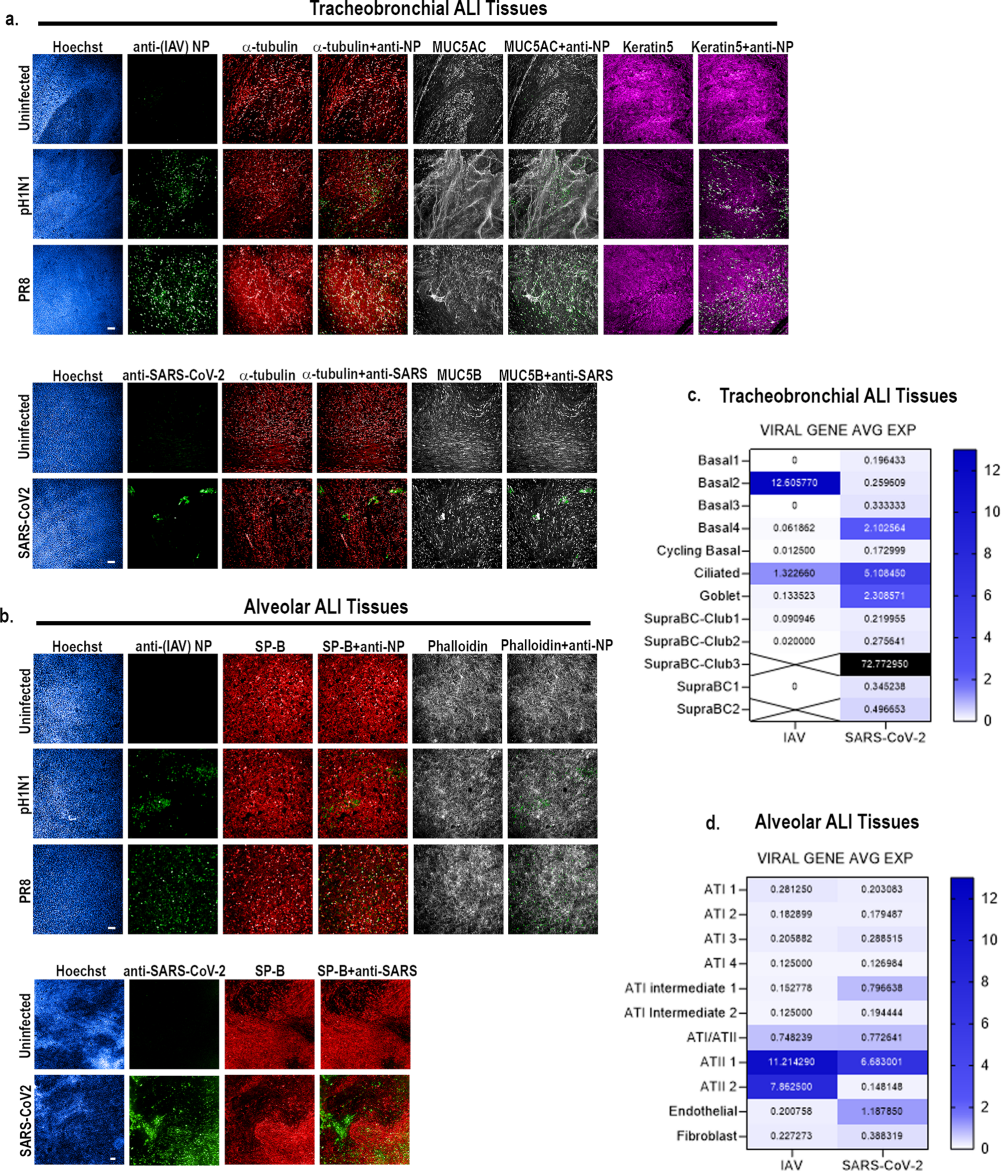
IAV and SARS-CoV-2 productively infect tracheobronchial and alveolar ALI tissue equivalents. Tracheobronchial and alveolar ALI tissues were infected with IAV strains pH1N1 or PR8 (1x10e5 TCID_50_ units), or SARS-CoV-2 (1x10e5 TCID_50_ units), (*n* = 3). Infected tissues were fixed at 24 hpi for IAV inoculated tissue, 36 hpi for SARS-CoV-2 inoculated tracheobronchial ALI tissue, or 144 hpi for SARS-CoV-2 inoculated alveolar ALI tissue and stained with antibodies against selected cell markers and virus antigens as indicated: **a** Tracheobronchial ALI tissues were stained with anti-(IAV) NP (green, top three panels) or anti-SARS-CoV-2 (monoclonal antibody cocktail targeting S and N proteins, green, bottom two panels), anti-α-tubulin (ciliated cell marker, red), anti-MUC5AC or MUC5B (goblet cell markers, white), and anti-keratin 5 (basal cell marker, magenta). **b** Alveolar tissues were stained with anti-(IAV) NP (green top three panels) or anti-SARS-CoV-2 (green, bottom two panels) as the marker of infected cells as well as anti-surfactant protein B (SP-B, ATII cell marker, red), phalloidin (F-actin, general cell marker, white). Scale bar is 100 μm and 200 μm in IAV and SARS-CoV-2 infected tissues, respectively. Viral gene expression levels, expressed as log transform mean expression, in identified cell subpopulation types within the human (**c**) tracheobronchial or (**d**) alveolar ALI tissues after 48 h of infection with PR8-IAV (left column), or after 72 h of infection with SARS-CoV-2 (right column). Identified viral genes per cell type were averaged and plotted as average log mean expression.

We next determined whether SARS-CoV-2 and/or IAV can infect human alveolar and tracheobronchial ALI tissues by immunofluorescence and scRNAseq. In IAV-exposed tracheobronchial ALI tissues, we observed viral antigen presence within multiple cell types at 24 h post-infection (hpi), with basal cells (KRT5^+^) being the dominant infected cell type, followed by ciliated cells (α-tubulin^+^) and goblet cells (MUC5AC^+^) (Fig. 2a, Supplementary Fig. 1). Correspondingly, scRNA-seq data showed the highest IAV PR8 transcript levels at 48 hpi in basal cells (basal2 cells, 12.606 avg. expression), followed by ciliated cells (1.3226 avg. expression) and goblet cells (0.133523 avg. expression) (Fig. 2c). In SARS-CoV-2 infected tracheobronchial ALI tissues, we observed co-staining of SARS-CoV-2 N antigen with both ciliated (α-tubulin^+^) and goblet cells (MUC5B) 36 hpi (Fig. 2a; Supplementary Fig. 1), in agreement with the location of known SARS-CoV-2 host entry co-factors. We observed detectable (>0.1 average) expression of SARS-CoV-2 viral transcripts in every identified cell type; however, as expected based on antigen staining data, we observed high viral transcript levels in ciliated cells (avg. expression 5.108450), as well as in goblet cells (avg. expression 2.308571), and a subtype of basal cell (Basal4, avg. expression 2.102564) (Fig. 2c). Interestingly, we also identified a small population of a subgroup of suprabasal cells (SupraBC-Club3) that had remarkably high SARS-CoV-2 transcript levels (average expression 72.772950) (Fig. 2c).

In alveolar ALI tissues, the majority of cells infected by IAV or SARS-CoV-2 were positive for SP-B (Fig. 2b). In agreement, scRNAseq analysis revealed the highest IAV or SARS-CoV-2 transcript expression level in ATII cells (Fig. 2d. IAV: ATII 1 and ATII 2, avg. viral gene expression 11.214 and 7.863, correspondingly; SARS-CoV-2 ATII 1, avg. viral gene expression 6.683), indicating primary infection of ATII cells, although another SP-B negative cell subpopulation was also positive for SARS-CoV-2 N antigen (Fig. 2b). We also detected SARS-CoV-2 transcripts in endothelial cells in the ALI alveolar tissues (Fig. 2d; 1.188 avg. expression); however, we were unable to detect cells co-positive for the endothelial cell marker CD31 and SARS-CoV-2 N antigen at this time point. We also used module-scoring analysis, which subtracts the aggregated expression of randomly selected control feature sets from the average expression level of viral gene clusters on the single cell level, which confirmed dominantly infected cell types (Supplementary Fig. 2).

### Human tracheobronchial and alveolar ALI tissues exhibit slower viral infection kinetics for SARS-CoV-2 compared to IAV

**Fig. 3:**
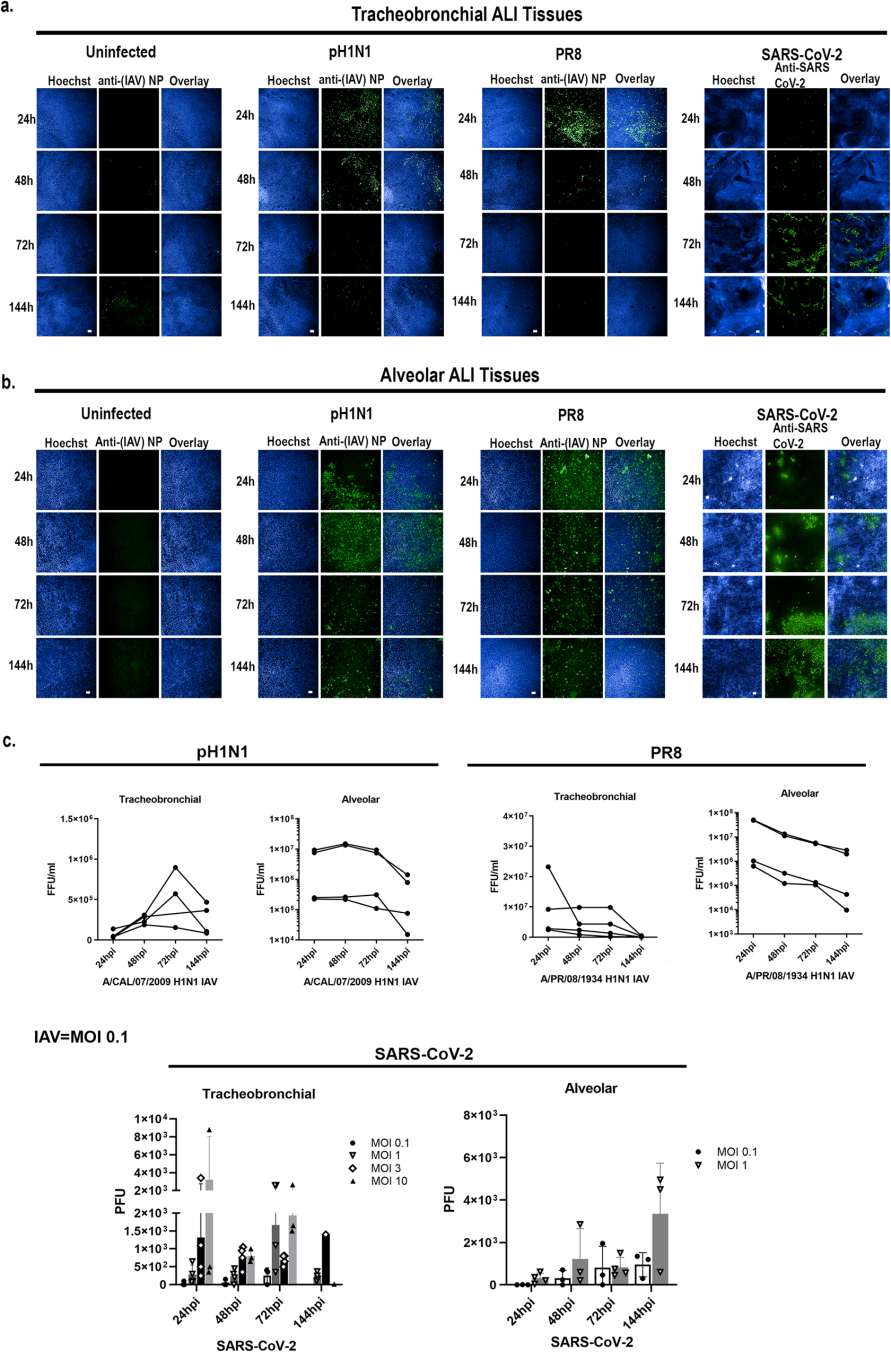
IAV and SARS-CoV-2 exhibit different infection kinetics in tracheobronchial and alveolar ALI tissue equivalents. Tracheobronchial and alveolar ALI tissues were infected with IAV pH1N1 or PR8 at approx. MOI of 0.1, and SARS-CoV-2 at MOI of 1 (fixed tissue samples shown) or as indicated in titer plots. Apical washes were collected and tissues fixed at 24, 48, 72 and 144 hpi. **a** Tracheobronchial and (**b**) alveolar ALI tissues were stained with anti-(IAV) NP and anti-SARS-CoV-2 to label infected cells (shown in green) as well as the nuclear dye Hoechst (blue). **c** Production of infectious virus from the apical chamber of tracheobronchial or alveolar ALI tissues after exposure to pH1N1 (MOI of ~0.1), PR8 (MOI of ~0.1), or SARS-CoV-2 (MOI of ~0.1 and ~1 for alveolar tissues; MOIs of ~0.1, ~1, ~3, and ~10 for tracheobronchial tissues) at 24, 48, 72, or 144 hpi. IAV titers were measured using a focus forming unit assay on LLC-MMK2 SIAT1 cells, SARS-CoV-2 was measured using plaque assay on Vero E6 cells and expressed as total FFU (IAV) or PFU (SARS-CoV-2)/tissue. Scale bar is 100 μm and 200 μm in IAV and SARS-CoV-2 infected tissues, respectively. Data are represented as M ± SD for a minimum of *n* = 3 independent experiments/biological replicates except for the data from SARS-CoV-2 infected tracheobronchial tissues at 144 hpi MOI = 3 and MOI = 10 that has *n* = 1.

Unlike IAV, which has a relatively short clinical incubation period of 1.5-2 days, SARS-CoV-2 has an average incubation period of 5.5 days after initial infection^49^. In agreement with this clinical observation, our data indicate that the number of IAV PR8 infected cells in both tracheobronchial and alveolar ALI tissues rapidly spiked at 24 hpi, followed by a gradual decline in both infected cells (Fig. 3a, b) and in secreted virus (Fig. 3c), in agreement with the robust and rapid replication described in the human host. Infection with IAV pH1N1 (MOI 0.1) resulted in the highest staining of IAV antigen at 48 hpi in both ALI tissues (Fig. 3a, b), followed by a decline in infectious virion production by 144 hpi (Fig. 3c). Conversely, SARS-CoV-2 replication in both tissues was slower and of less magnitude when compared to IAV (Fig. 3c), and in agreement with a longer incubation period in humans. Indeed, the number of SARS-CoV-2 N antigen positive cells in both ALI tissues was low at early time points but continued to increase steadily over time in infected ALI tissues independently of the MOI used (0.1 to 1), with tracheobronchial ALI tissues exhibiting peak infection at 72 hpi (Fig. 3a) and alveolar ALI tissues at the latest time point tested (144 hpi) (Fig. 3b). Correspondingly, infectious virion production from SARS-CoV-2 infected alveolar ALI tissues peaked at 144 hpi (Fig. 3c). In tracheobronchial ALI tissues, viral production was also dose-dependent, although infection with higher MOIs (3 and 10) resulted in a higher virion production at 24 hpi, followed by a decline at later time points, which may be due to cell death as a result of an initially high viral exposure (Fig. 3c). Interestingly, alveolar ALI tissues seemed to be more susceptible to IAV and SARS-CoV-2 infection than tracheobronchial ALI tissues, as evidenced by the higher number of infected cells (Fig. 3b) and viral titers (Fig. 3c). Cellular markers for each time-point are shown in Supplementary Fig. 3. Thus, both IAV and SARS-CoV-2 can productively infect lung ALI tissues, though with different kinetics that may contribute to their different clinical incubation periods.

**Fig. 4:**
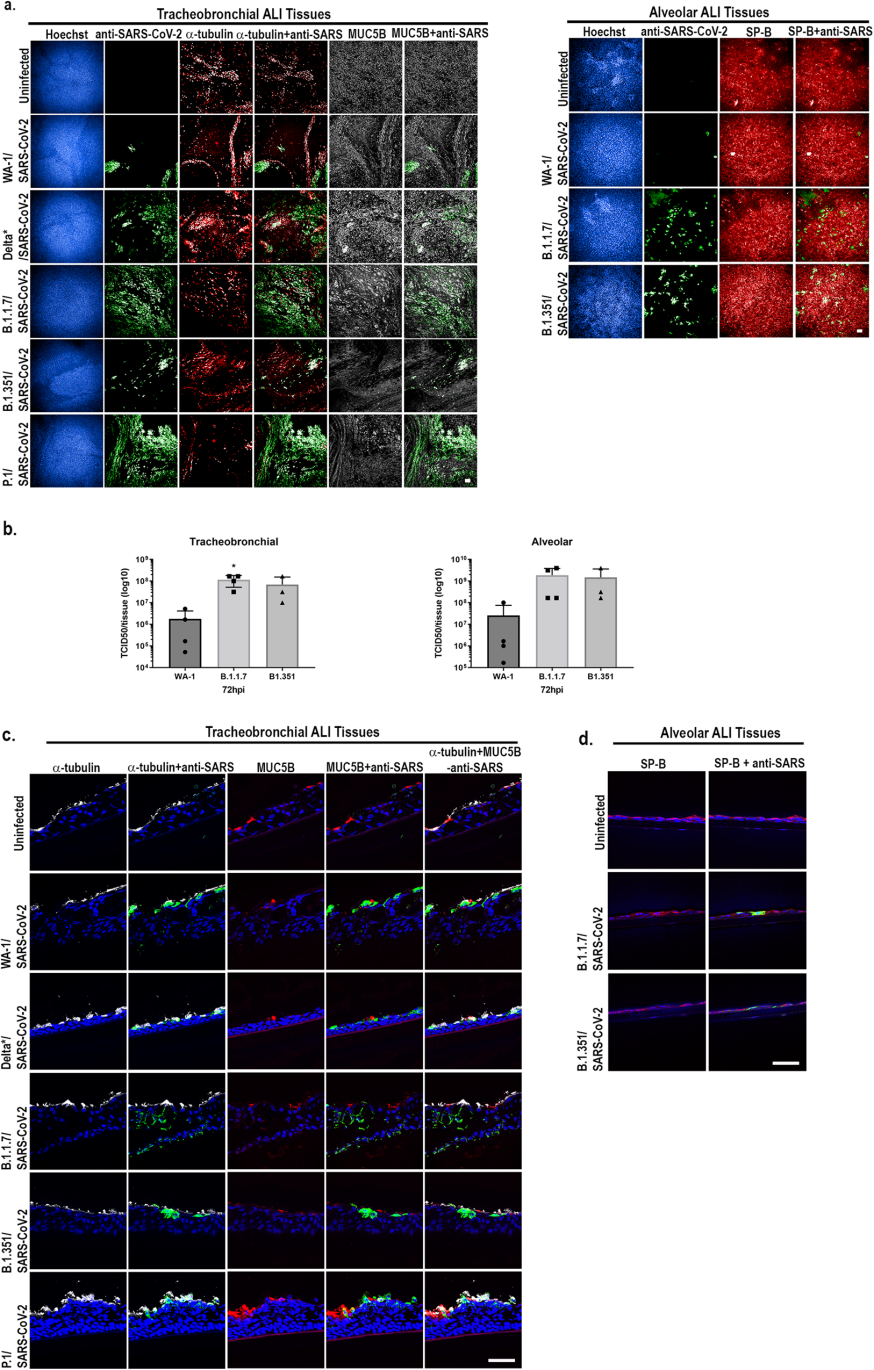
Infection of alveolar and tracheobronchial ALI tissues with SARS-CoV-2 VoC exhibit enhanced infection. Tracheobronchial and alveolar ALI tissues were infected with each SARS-CoV-2 VoC at 2×10^5^PFU/tissue. Apical washes were collected at 72, 96, and 144 hpi and tissues fixed at 72 hpi (tracheobronchial) or 96 hpi (alveolar). **a** Tracheobronchial and alveolar ALI tissues were stained with anti-SARS-CoV-2 to label infected cells (shown in green) as well as anti-tubulin (red) and anti-MUC5B (white), or anti-SP-B (red) and the nuclear dye Hoechst (blue). Scale bar is 100 μm. **b** Production of infectious virus from the apical chamber of tracheobronchial or alveolar ALI tissues after exposure to SARS-CoV-2 at 72 hpi. SARS-CoV-2 was measured using TCID_50_ assay on Vero-TMPRSS2 cells, and expressed as total TCID_50_/tissue. The 63x confocal images of cryosectioned (10 μm thickness) SARS-CoV-2 VoC infected tracheobronchial (**c**) or alveolar (**d**) tissue slices stained with anti-SARS-CoV-2 to label infected cells (shown in green) as well as (**c**) anti-tubulin (red) and anti-MUC5B (white), or (**d**) anti-SP-B (red) and the nuclear dye Hoechst (blue). Data are represented as M ± SD for a minimum of *n* = 3 independent experiments/biological replicates. Scale bar is 50 μm.

Additionally, to investigate whether we could replicate differences in infectivity and viral production observed in vitro and in vivo between SARS-CoV-2 variants^50–52^, we infected tracheobronchial or alveolar ALI tissue with an approximate MOI of 0.2-0.4 (2 × 10^5^ PFU/tissue) and then stained fixed tissues for detection of SARS-CoV-2 NP or measured viral production at various time points post-infection. We observed increasing viral NP antigen (Fig. 4a) as well as increased viral production (Fig. 4b) after infection with different VoC, including B.1.1.7 and B.1.351, compared to the WA-1 variant at 72 hpi or 96 hpi in tracheobronchial and alveolar ALI tissues. Results also showed similar cellular tropism by the viral variants studied (Fig. 4c, d).

### Human tracheobronchial and alveolar ALI tissues exhibit distinct transcriptomic profiles in response to IAV or SARS-CoV-2 infection

**Fig. 5:**
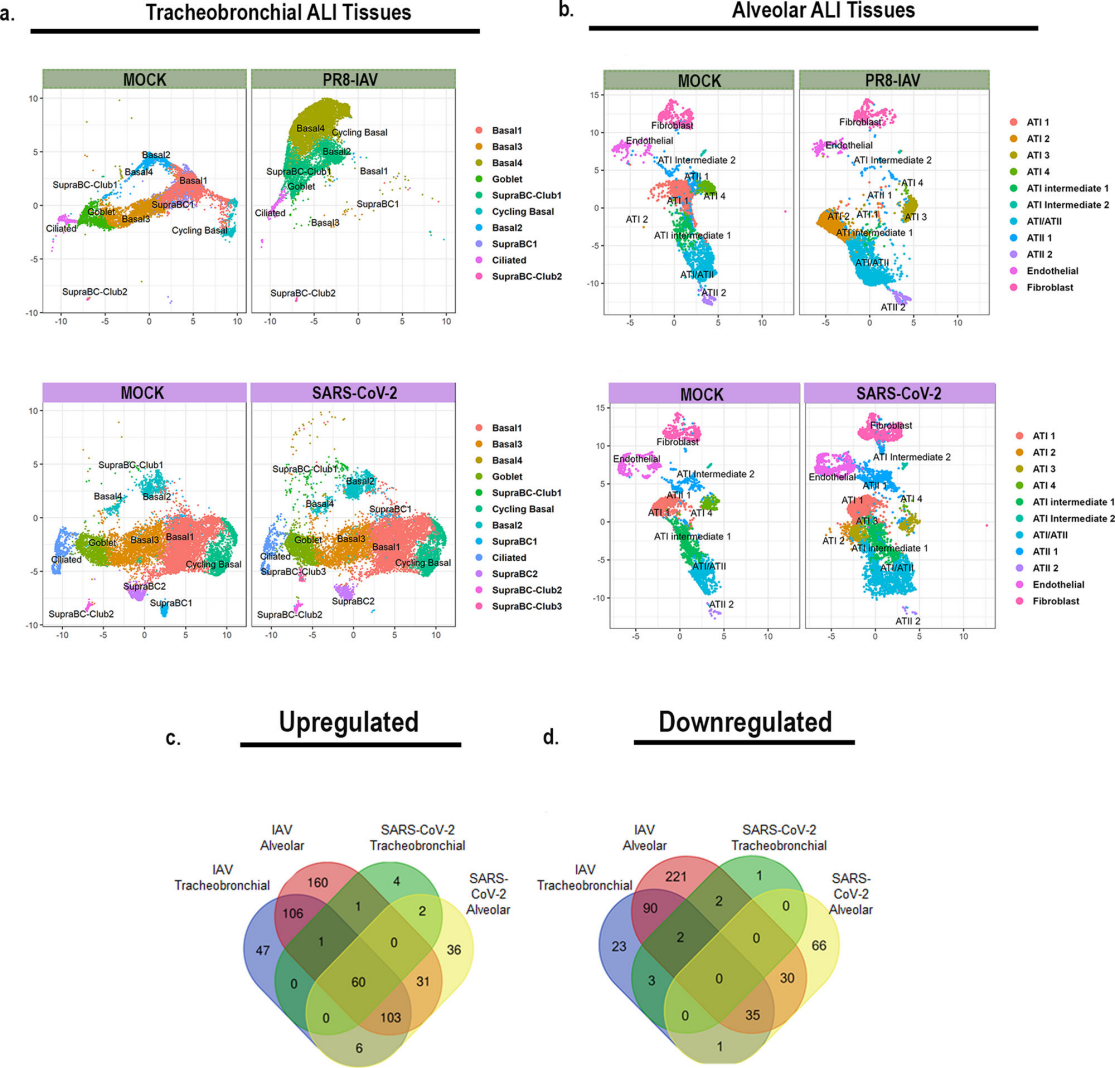
ScRNAseq UMAP plots of IAV or SARS-CoV-2 infected lung ALI tissues. Tracheobronchial and alveolar ALI tissues were infected with PR8 IAV (1e5 TCID50 units/tissue, *n* = 2) for 48 h or SARS-CoV-2 (2e5 PFU/tissue, *n* = 2) for 72 h prior to dissociation for scRNAseq. Shown here are UMAP plots of: (**a**) Tracheobronchial tissues infected with mock media or PR8 IAV for 48 h (top, green), or with mock media or SARS-CoV-2 for 72 h (bottom, purple). **b** Alveolar tissues infected with mock media or PR8 IAV for 48 h (top, green), or with mock media or SARS-CoV-2 for 72 h (bottom, purple). Venn diagrams showing viral and tissue unique or shared genes with Log2FC values (**c**) >0.264 or (**d**) <-0.264 over mock in IAV or SARS-CoV-2 infected airway and alveolar tissues.

We next sought to elucidate cell-specific and tissue-wide transcriptomic responses to viral infection in the context of the two lower respiratory tract ALI tissue models. To do this, we analyzed single cell gene expression shifts by Uniform Manifold Approximation and Projection plots (UMAP). Interestingly, while we observed a dramatic, tissue-wide shift in the number of cells in each of the cell type populations for IAV infected tracheobronchial ALI tissues as well as alveolar ALI tissues (Fig. 5a, b, top panels), regardless of actual individual cell infection status, we observed a much more subtle shifts in each cell type in SARS-CoV-2 infected tissues (Fig. 5a, b bottom panels). Volcano plots indicate major (log2fc = 0.7, *p* value threshold <0.05) gene expression shifts in both ALI tissues (mock infected vs. 48 hpi PR8-IAV or 72 hpi SARS-CoV-2 infected) (Supplementary Fig. 4). Interferon stimulated gene 15 (*ISG15*), an ubiquitin-like protein that plays a multifaceted role in viral infections and viral induced inflammation, was among the most strongly upregulated in all four tested conditions^53,54^. Of the top 20 upregulated genes upon viral infection (IAV or SARS-CoV2), in both ALI tissues, 11/20 genes were shared (*IFI27*, *ISG15, IFI6, IFIT1, BST2, IFIT2, IFIT3, CXCL10, MX1, OAS1*, and *IFI44L*), with a primary function as defense response to viral infection (DAVID Analysis, GOTERM_BP_DIRECT, GO:0051607: defense response to virus, 10/11 genes, *p* value 2.9e-17). DEG analysis revealed a higher number of modulated genes in IAV infected ALI tissues (477 and 838 total DEGs in tracheobronchial and alveolar ALI tissues, respectively) compared to SARS-CoV-2 infected ALI tissues (76 and 370 total DEGs in tracheobronchial and alveolar ALI tissues, respectively). (Fig. 5c, d; Supplementary Data 1, tabs 1 and 2). In general, alveolar ALI tissues showed a higher number of DEGs after infection with both viruses.

**Fig. 6:**
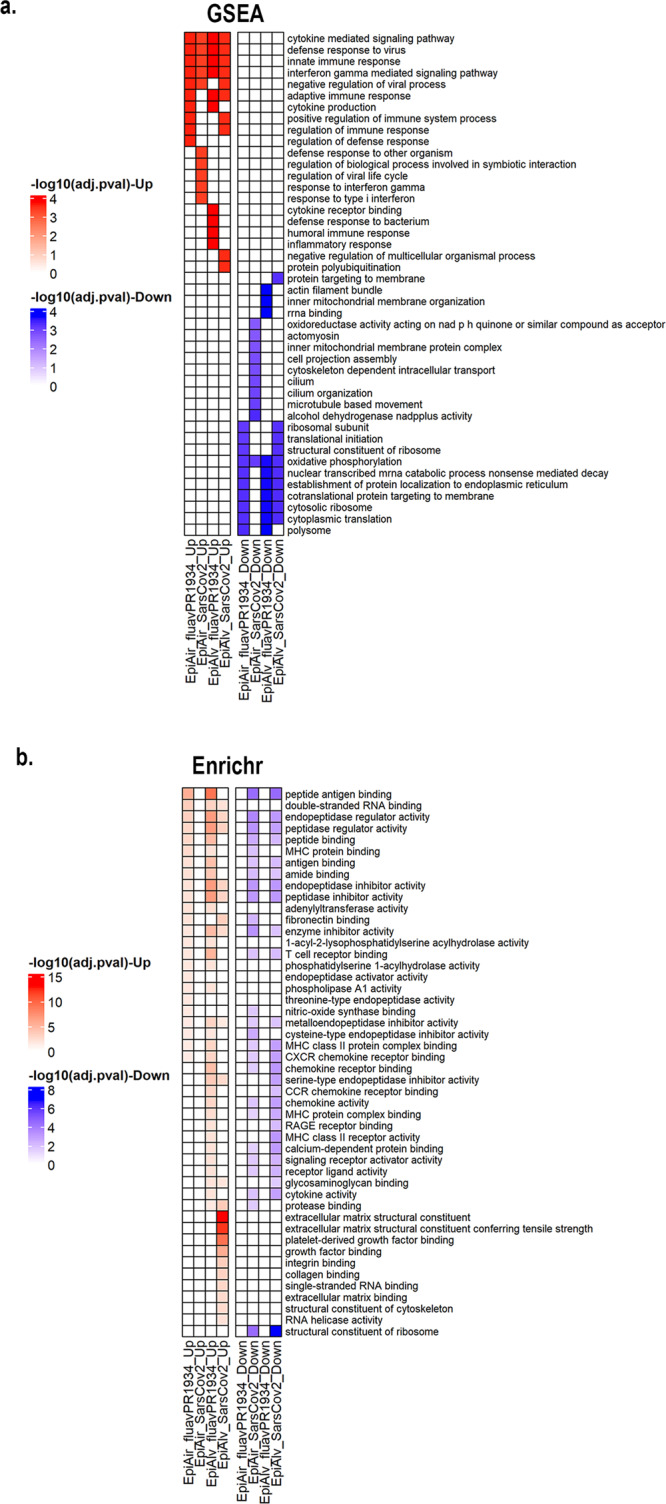
Pathway enrichments per ALI tissue per virus. **a** Heatmap of top 10 up- (red) or down-regulated (blue) pathways by GSEA, expressed as -log10 adjusted *p* value; (**b**) heatmap of top 20 differentially regulated pathways between the four conditions by Enrichr analysis, expressed as -log10 adjusted *p* value.

We observed 60 upregulated genes common to both IAV and SARS-CoV-2 infected tracheobronchial and alveolar ALI tissues, while no shared downregulated genes were observed across both ALI tissues and/or viruses (Fig. 5c, d, Supplementary Data 1). Gene set enrichment analysis (GSEA) revealed general shared antiviral responses, including upregulated cytokine mediated signaling pathway (GO:0019221), defense response to virus (GO: 0051607), innate immune response (GO:0045087) and the IFN-γ mediated signaling pathway (GO:0060333), part of the top ten enriched pathways in the both tissue types in response to IAV and SARS-CoV-2 infection (Fig. 6a, Supplementary Data 2). Other pathways upregulated in some of the tissues and/or viral infections are adaptive immune responses, cytokine production, response to type I IFN and inflammatory responses. Downregulated pathways include oxidative phosphorylation, cytosolic ribosome and cytoplasmic translation, mitochondrial organization and microtubule movement, among others (Fig. 6a).

We next compared our four conditions (IAV *vs*. SARS-CoV-2 in tracheobronchial *vs*. alveolar ALI tissues) and looked at divergent gene pathway analysis by Enrichr analysis using an absolute log2fc of 0.264 as a cut-off (Fig. 6b, Supplementary Figs. 5–7). Interestingly, MHC protein binding and peptide antigen presentation pathways, both critical for T-cell responses to viral infections^55^, were highly enriched in IAV infected tracheobronchial and alveolar ALI tissues, but mostly downregulated in SARS-CoV-2 infected ALI tissues (Fig. 6b). For Enrichr identified pathways, we mapped both tissue wide (Supplementary Figs. 6–7) and responding cells by cell identity type and infection status to pathway enrichment in the infected ALI tissues. In general, while we observed differential responses between cell types, both infected and non-infected cells in the virus exposed ALI tissue responded similarly if identified as a similar cell type. Future work will further characterize cell-type specific responses and the role of cell-cell signaling in the viral disease state of each ALI tissue.

### Human tracheobronchial and alveolar ALI tissues exhibit distinct inflammatory cytokine profiles in response to IAV or SARS-CoV-2 infection

To determine this tissue-specific inflammatory response, we measured secreted proteins from SARS-CoV-2 and IAV infected ALI tissues, as well as determined cytokine/chemokine production by specific cell types by scRNAseq of single time point of infection.

**Fig. 7:**
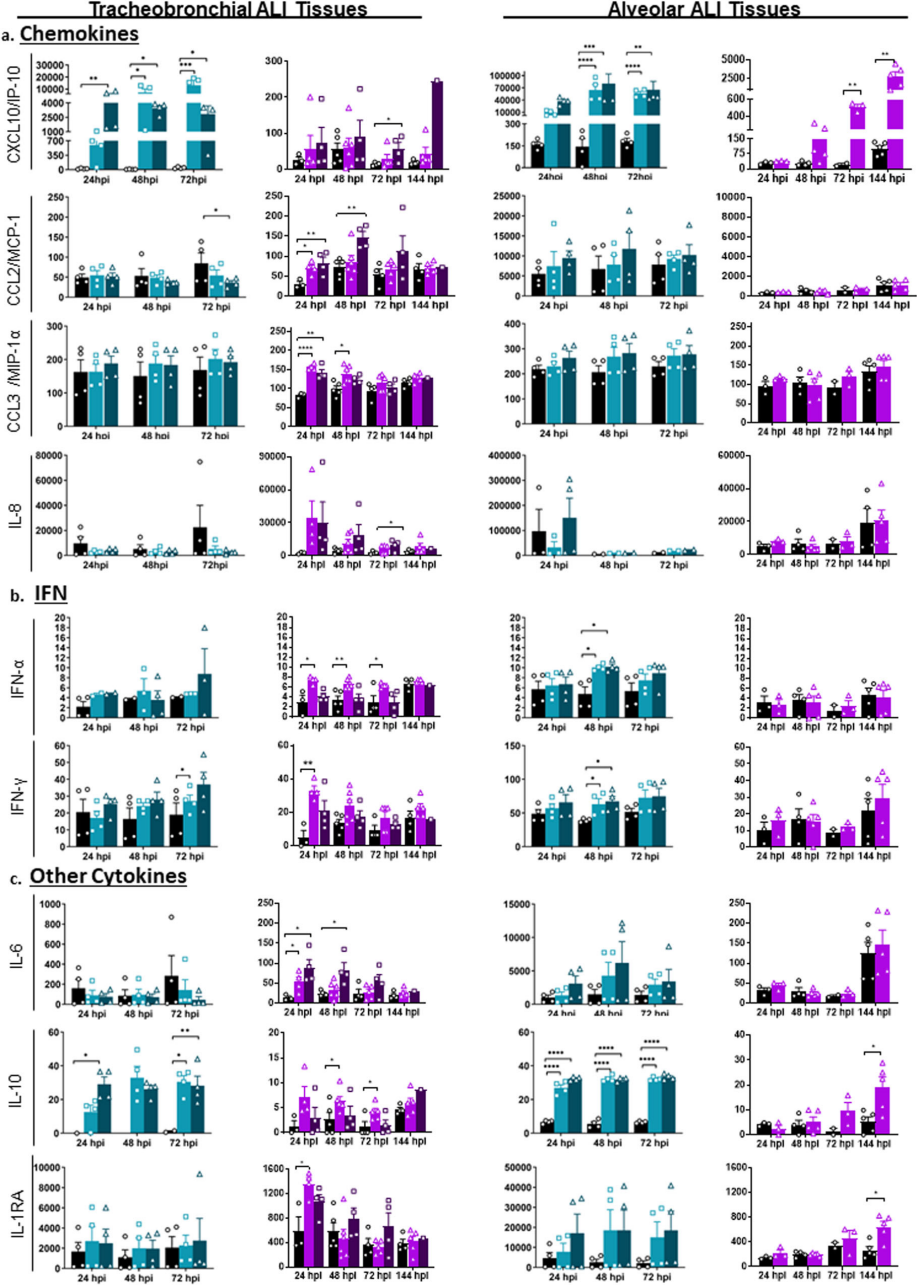
Alveolar and tracheobronchial ALI tissues produce tissue-specific chemokines and cytokines in response to IAV and SARS-CoV-2 infection. Basal compartment media were collected from tracheobronchial (left two panels) or alveolar (right two panels) ALI tissues at indicated time-points and analyzed for cytokine and chemokine secretion by a custom Luminex assay: (**a**) Chemokines (CXCL10/IP-10, CCL2/MCP-1, CCL3/MIP-1α, IL-8); (**b**) Interferons (IFN-α and IFN-γ); and (**c**) Other cytokines (IL-6, IL-10, IL-1RA). IAV infected tissues (MOI of ~0.1) are represented in shades of teal, where light teal shows infection with the IAV pH1N1 strain and dark teal shows infection with the IAV PR8 strain, whereas SARS-CoV-2 infected tissues are represented in shades of purple, with progressing color from low MOI (~1) to high MOI (~10). All measurements on y axis are in pg/ml. Data are represented as M ± SEM for a minimum of *n* = 3 independent experiments and/or biological replicates except for the data from SARS-CoV-2 infected tracheobronchial tissues at 144 hpi MOI = 10 that has *n* = 1 and the SARS-CoV-2 set matched Mock alveolar tissues at 72hpi that has *n* = 2; Student *t* test of IAV or SARS-CoV-2 infected tissues *vs*. uninfected controls at each timepoint: *p  <  0.05, **p  <  0.005, ***p  <  0.0005, ****p  <  0.00005.

Tissues infected with IAV showed an earlier and stronger immune response. Chemokine CXCL10/IP-10 was significantly increased in both tracheobronchial and alveolar ALI tissues infected with either pH1N1 or PR8 IAV strains, showing an early and robust response (Fig. 7) that correlated with maximum viral production (Fig. 3c). *CXCL10/IP-10* was highly expressed in SupraBC-Club, cycling basal and ciliated cells (tracheobronchial ALI tissue) and ATI and ATII cells (alveolar ALI tissue) (Supplementary Fig. 9). Chemokine production after SARS-CoV-2 infection appeared to be mostly restricted to tracheobronchial ALI tissues, with significantly higher protein production at either early (CCL2/MCP-1 and CCL3/MIP-1α) or later (CXCL-8/IL-8 and CXCL-10/IP-10, both produced mostly by SupraBC-Club and basal cells, Supplementary Fig. 9) time points (Fig. 7a). CXCL-10/IP-10 was the only chemokine induced in SARS-CoV-2 infected alveolar ALI tissues (Fig. 7a), correlated with increased infectious viral particles at later time points (Fig. 3c). Elevated chemokine levels observed are in agreement with the chemokine storm observed in severe human COVID-19 cases and different SARS-CoV-2 experimental models^56,57^, where the role of CXCL-10 has been particularly highlighted in COVID-19-associated Acute Respiratory Distress Syndrome (ARDS) as well as in severe influenza^58^.

We next examined Th1, Th2, and Th17 immune responses, including type I (IFN-α and IFN-β), type II (IFN-γ), and type III (IFN-λ) interferons. We observed a modest but significant type I (IFN-α) and II (IFN-γ) interferon response in some of the IAV (alveolar and tracheobronchial ALI tissues) and SARS-CoV-2 (tracheobronchial ALI tissue) infected ALI tissues compared to the uninfected controls (Fig. 7b). IFN-β was either not significant (IAV infected ALI tissues) or not detected (SARS-CoV-2 infected ALI tissues), and IL-28A/IFN- λ2 protein levels were also below the limit of detection.

In addition, several Th1 (TNF-α, TNF-β), Th2 (IL-18), and Th17 (IL-17) markers were significantly elevated or trended upwards in at least one IAV infected ALI tissue compared to uninfected controls (Supplementary Fig. 8), demonstrating a modest but sustained inflammatory response to influenza, especially in the alveolar ALI tissue. The cytokine response to SARS-CoV-2 infection was mostly observed in tracheobronchial ALI tissues (Supplementary Fig. 8). Only TNF-α and IL-18 (Supplementary Fig. 8), the latter produced by different cell types (Supplementary Fig. 9), showed moderate but significant increased levels in alveolar ALI tissues after 72 hpi, in correlation with maximum viral production (Fig. 3c). IL-1β was not detected during SARS-CoV-2 infection.

We also measured secreted IL-6, associated with the acute inflammatory response in COVID-19 and influenza, as well as the increased production of anti-inflammatory IL-10, which maintains immune homeostasis^59–62^. The presence and ratio of IL-6 and IL-10 may be used as predictors of COVID-19 disease severity. IL-6 was increased in SARS-CoV-2 infected tracheobronchial ALI tissues at early time points, but only showed an increased trend in IAV infected alveolar ALI tissues. However, IL-10 was higher in both ALI tissues infected with either SARS-CoV-2 (starting at 48 hpi) or IAV (at all time points) (Fig. 7c). In addition, anti-inflammatory IL-1RA (Fig. 7c) was significantly higher at some time points after infection with SARS-CoV-2 (expressed by *IL-1RN* gene in SupraBC-Club and basal cells in tracheobronchial ALI tissues, and ATI and ATII cells in alveolar ALI tissues, Supplementary Fig. 9).

Lastly, we observed some increased production of other immune markers such as G-CSF (neutrophil development and function), EN-RAGE/S100A12 (migration and recruitment of leukocytes), IL-7, and hepatocyte growth factor (HGF) in response to IAV and/or SARS-CoV-2 up to 72 hpi (Supplementary Fig. 8). These have been found elevated in the serum of hospitalized COVID-19 patients^63,64^, and are correlated with COVID-19 disease severity. A dot map of the log2fc of other detected chemokine/cytokine genes for each cell type identified by scRNAseq in the tracheobronchial or alveolar ALI tissues before and after infection is presented in Supplementary Fig. 8.

### Validation of antiviral drugs in lung tissue equivalents

**Fig. 8:**
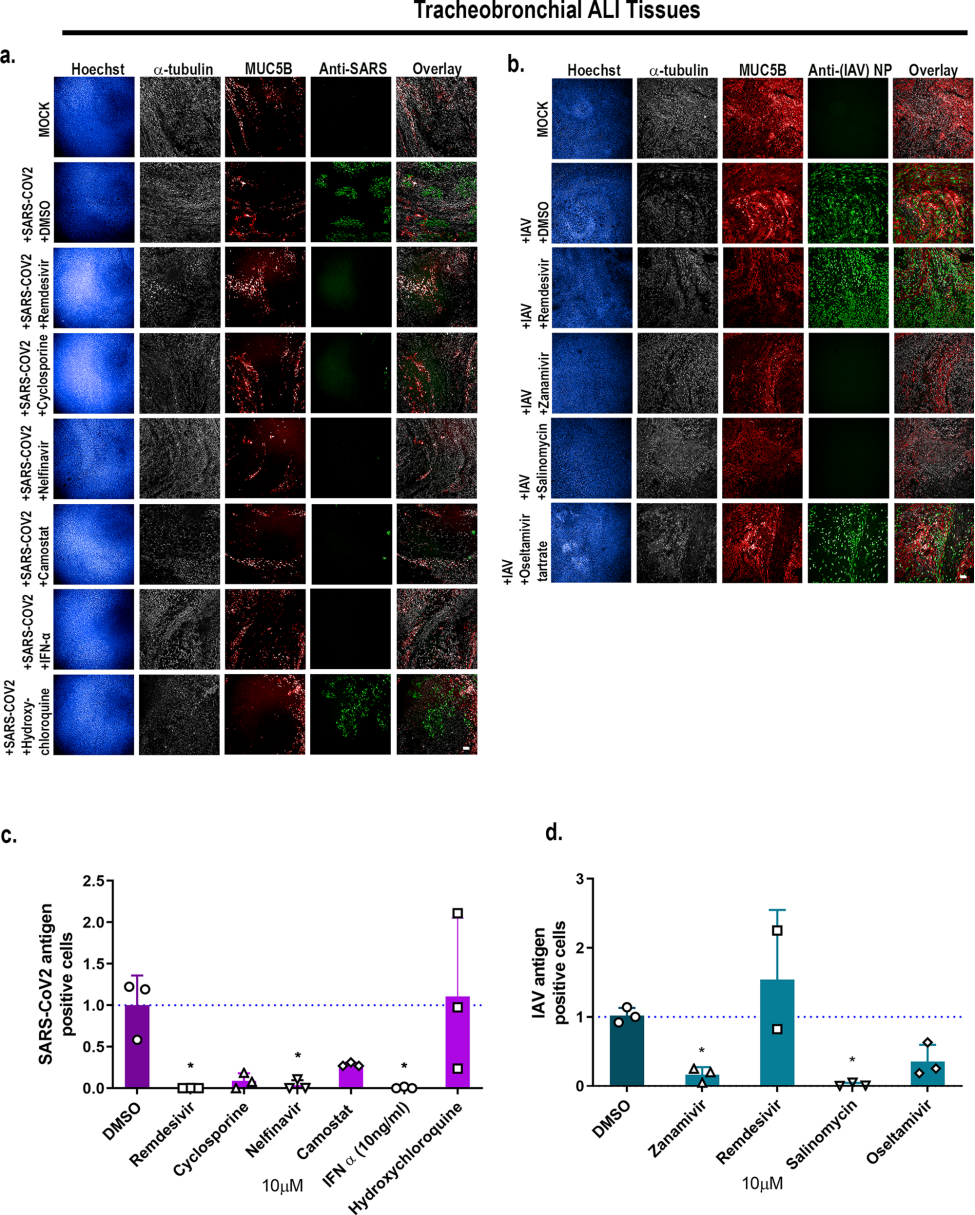
Tracheobronchial and alveolar ALI tissue equivalents predictively measure antiviral compound response in the context of SARS-CoV-2 and IAV infections. Selected compounds were added to the basal media chamber (10 μM final concentration) of the tracheobronchial and alveolar ALI tissues for 1 h and then infected with IAV PR8 (MOI of 0.1), and SARS-CoV-2 (MOI of 0.1). IAV and SARS-CoV-2 infected tissues were fixed for 24 and 36 hpi, respectively, and stained with antibodies against selected cell markers and viral specific antigens. **a**, **b** Tracheobronchial ALI tissues were stained with anti-α-tubulin (ciliated cell marker, white), anti-MUC5AC or anti-MUC5B (goblet cell marker, red), along with anti-N protein (green, right five panels) and anti-SARS-CoV-2 (monoclonal antibody cocktail targeting S and N proteins, green) as the marker of infected cells Scale bar is 100 μm. Image-based quantification of infected cells in compound treated and subsequent (**c**) SARS-CoV-2 or (**d**) IAV infected tracheobronchial ALI tissues. Data are represented as M ± SD for a minimum of *n* = 3 independent experiments and/or biological replicates except for the data from Remdesivir treated IAV infected tissues that has *n* = 2; Student *t* test of IAV or SARS-CoV-2 infected tissues *vs*. uninfected controls at each timepoint: *p  <  0.05, **p  <  0.005, ***p  <  0.0005, ****p  <  0.00005.

To evaluate the ability of these physiological relevant ALI tissues to measure antiviral drug activity, we tested a panel of antiviral drugs on either tracheobronchial or alveolar cultures for antiviral activity. We first tested remdesivir, which is granted for emergency use authorization by the US Food and Drug Administration (FDA) for hospitalized COVID-19 patients^65^, and type I interferon (IFN-β) for anti-SARS-CoV-2 activity. As expected, both remdesivir and IFN-β robustly inhibited SARS-CoV-2 infection as seen by qRT-PCR and direct SARS-CoV-2 antigen staining in the tracheobronchial ALI tissues (Fig. 8, Supplementary Fig. 10). Furthermore, we tested two other compounds: cyclosporine, previously identified in a high-throughput screen with anti-SARS-CoV-2 infection activity in both human hepatoma (Huh7.5 cells) and human lung adenocarcinoma cells (Calu-3) in a monolayer cell-based model^12^, as well as hydroxychloroquine. Neither cyclosporine nor hydroxychloroquine reduced SARS-CoV-2 infection in the tracheobronchial lung model (10 ${{{{{\rm{\mu}}}}}}$M, Fig. 8a, bottom two rows, Fig. 8c, Supplementary Fig. 10a, c). Furthermore, hydroxychloroquine failed to reduce SARS-CoV-2 viral production in the alveolar model (10 ${{{{{\rm{\mu}}}}}}$M, Supplementary Fig. 10d). However, when we tested camostat, a TMPRSS2 inhibitor, and nelfinavir, an anti-retroviral, currently being tested in clinical trials^40,66–69^, both reduced SARS-CoV-2 infection in the tracheobronchial ALI tissue (Fig. 8a, c, Supplementary Fig. 10a, 10 ${{{{{\rm{\mu}}}}}}$M) and alveolar ALI tissues (Supplementary Fig. 10b, 10 ${{{{{\rm{\mu}}}}}}$M).

We next tested whether anti-SARS-CoV-2 compounds remdesivir and nelfinavir would also reduce IAV infection in the tracheobronchial (Fig. 8b) or alveolar (Supplementary Fig. 11) ALI tissues. Neither remdesivir nor nelfinavir reduced IAV antigen staining in these tissues. However, clinically approved anti-IAV compounds zanamivir and oseltamivir, as well as the reported anti-IAV drug salinomycin, did inhibit IAV infection using a single dose approach in the tracheobronchial (Fig. 8b, d) ALI tissues at 10 ${{{{{\rm{\mu}}}}}}$M. Overall, these findings validate the use of ALI tissues for studying the efficacy of anti-viral drugs.

## Discussion

We have shown that both primary human tracheobronchial and alveolar ALI tissues in a transwell plate format are relevant 3D in vitro models for studying multiple aspects of SARS-CoV-2 and IAV infections, in addition to being a valuable platform for antiviral drug testing. We chose to investigate two distinct LRT models, representing the distal airway (tracheobronchial) and proximal airway (alveolar). The tracheobronchial region of the airway is made of a pseudostratified epithelium comprised of ciliated, goblet, columnar club, and basal cells, whereas the alveolar region is comprised of squamous alveolar type I cells and cuboidal alveolar type II cells^70^. Whereas mucus secretion and mucociliary clearance occurs in the proximal airway, gas exchange primarily occurs in alveoli. Progression of infection from the upper respiratory tract to the lower respiratory tract, and from the distal to the proximal lung is necessary for both severe COVID-19 and influenza disease. Samples taken from patients with fatal COVID-19 have shown infection in ciliated bronchiolar epithelial cells, ATIIs, goblet cells, club-like cells, and endothelial cells^71–73^. Similarly, we saw SARS-CoV-2 infection peaking at 3-6 dpi, of both ciliated (α-tubulin^+^) and goblet (MUC5AC^+^) cell populations in the tracheobronchial ALI tissues, as well as ATs in the alveolar ALI tissues. We also observed robust SARS-CoV-2 and IAV infectious viral production in both ALI tissues, although apical washes collected from alveolar ALI tissues exhibited slightly higher SARS-CoV-2 titers than tracheobronchial ALI tissues (Fig. 3). In contrast, a previously reported lung-on-chip model, consisting of primary human ATs and human lung microvascular endothelial cells, did not find productive infection with SARS-CoV-2 in ATs^74^. This may be due to differences in receptor expression in patient cells used for each study, including the absence of ACE2 expression in ATs in the lung-on-chip model compared to robust hACE2 expression in the alveolar ALI tissues in our study. While we did not observe SARS-CoV-2 N antigen in endothelial cells in our alveolar ALI tissue study, we detected SARS-CoV-2 viral RNA in endothelial cells. In this regard, future studies will need to look at viral infection from different patient-derived epithelial cells and over an extended period to characterize further the full infection dynamics in the tracheobronchial and alveolar ALI tissues, in addition to direct exposure of lung endothelial cells to SARS-CoV-2.

Comparison of the expression levels of SARS-CoV-2 putative receptors confirmed the low levels of expression of hACE2 (~1% of cells) which has been seen in human lung transcriptomics analysis^75^, which is in contrast with a robust detection hACE2 protein by antibody-based detection. Differences may be explained by long half-lifetime of proteins in the cellular membrane. Detection of viral transcripts in the cells of infected tissues highlighted infectivity that corresponded to that seen in histological samples for SARS-CoV-2. In addition, while we observed shared activation of certain cellular antiviral responses for both IAV and SARS-CoV-2 by scRNA-seq, including inflammatory and interferon-related anti-viral responses upregulated for both viruses and tissues similar to previous reports^76^, the intensity of the response, as measured by relative gene expression, differed between viruses and tissues, with IAV infection generally correlated with a stronger induction of antiviral related genes at 48 hpi, compared to the 72 hpi time point investigated for SARS-CoV-2. We observed select biological processes like antigen presentation, RAGE pathway activation, and downregulation of ribosomal biogenesis that were differentially modulated by these two viruses. A recent study which utilized RNAseq revealed that MHC class I genes were downregulated in SARS-CoV-2 human nasopharyngeal samples^77^. Similarly, we observed a significant downregulation of peptide antigen presentation pathway in the ALI airway tissues after infection with SARS-CoV-2, in contrast to an upregulation of peptide antigen presentation related genes and MHC Class I genes in IAV infected airway and alveolar tissues. The difference in antiviral response at the tested timepoints as well as the differential induction of pathways may explain different clinical pathological outcomes caused by the two viral infections.

Circulating chemokines, interferons, interleukins, growth factors and other pro-inflammatory cytokines are the main molecules involved in the development of the cytokine storm associated with COVID-19 severity. In particular, high levels of IL-6 and TNF are strongly associated with increased mortality, and elevated levels of anti-inflammatory cytokines IL-10 and IL-1RA have also been correlated with disease severity and fatal outcome^1,59,60,78–80^. However, circulating cytokines observed in serum may not represent local tissue cytokine levels, which may be key potentiators of the systemic hyperinflammatory response. In this regard, there have been extensive investigation into immunomodulatory drugs for the treatment of COVID-19^81^. In agreement with previous COVID-19 reports, we did find a significant induction of IP-10/CXCL10 levels in both tracheobronchial and alveolar SARS-CoV-2 infected ALI tissues (up to 25-fold increase in alveolar ALI tissues compared to uninfected controls), as well as other important chemokines and growth factors (CCL2/MCP-1, CCL3/MIP-1α, CXCL-8/IL-8, G-CSF) that were elevated in tracheobronchial ALI tissues. Interferon (IFN-α and IFN-γ) and Th1, Th2, and Th17 cytokine responses were only moderate but significant, and mostly restricted to tracheobronchial ALI tissues. It included pro-inflammatory (IL-6 and TNF-α), and anti-inflammatory (IL-10 and IL-1RA) immune modulators, known to have key roles in the pathogenesis associated to COVID-19 disease^1,59,60,78,79^. The cytokine induction after SARS-CoV-2 infection appeared to be tissue-dependent in our ALI models. Interestingly, even though alveolar ALI tissues had a more robust infection compared to tracheobronchial ALI tissues (as shown by the higher viral loads reported in Fig. 3), the cytokine and chemokine production was not as strong when compared to the overall response observed in tracheobronchial ALI tissues. Still, both ALI tissues had an overall immune response that corresponded with the infection dynamics, with alveolar ALI tissues showing slight increases in some cytokines at later time points (72 to 144 hpi) compared to earlier responses (24 to 72 hpi) in the tracheobronchial ALI tissues. In addition, scRNAseq analysis confirmed a suppressed IFN mediated response to SARS-CoV-2 infection in both tissues compared to IAV infection, while maintaining an increased chemokine induction. We also observed an inhibition of MHC Class I related peptide presentation in SARS-CoV-2 infected tissues, but not in IAV infected tissues, both supported by previous reports^76,77^.

A major limitation of these models is that we were only able to study the local epithelial-driven response by itself, without the contribution of myeloid cells or lymphocytes, which are important in mounting an appropriate innate immune response against viral infections. Still, we observed that both tracheobronchial and alveolar ALI tissues are capable of mounting a local epithelial-driven response to IAV and SARS-CoV-2 virus infection. In addition, the tracheobronchial ALI tissues did not contain pulmonary endothelial cells or pulmonary fibroblasts, unlike the alveolar ALI tissues however, we were able to map the cellular contributions by single cell sequencing (Supplementary Fig. 9). Future work will investigate the cross-talk between these cell types in initiating the epithelial response. Although myeloid cells were not included in this model, we hypothesize that the addition of the myeloid compartment in both tissues will show a more pronounced immune response that may reflect more accurately the different stages of human COVID-19 and severe influenza disease progression. Future studies will address the contribution of the lung epithelium and its role in recruiting additional inflammatory immune cells.

While there have been intense high-throughput screening efforts to discover potential antivirals for SARS-CoV-2, relatively few compounds have proven to be effective in clinical settings. Most of the studies published have relied on traditional mono-cellular tissue culture models, which do not allow crosstalk among different cell populations. In some cases, relying on in vitro activity profiles may prove detrimental. This is the case for hydroxychloroquine, initially shown to have anti-SARS-CoV-2 activity in vitro, but later proven ineffective at reducing COVID-19 patient outcome or hospitalization stay^24,25^. Interestingly, hydroxychloroquine failed to reduce SARS-CoV-2 infection in our in vitro ALI tissue equivalents, indicating that these 3D models might mimic better human tissue responses than other in vitro systems.

A primary human cell-based lung ALI tissue model may be a promising platform for newly emerged viral pathogens. Since the emergence of SARS-CoV-2/WA/2020, there have been seven lineages of variants identified as VoC, including Alpha (B.1.1.7 and Q lineages), Beta (B.1.351 and descendent lineages), Gamma (P.1 and descendent lineages), Epsilon (B.1.427 and B.1.429), Delta (B.1.617.2 and AY lineages) and Omicron (B.1.1.529 and BA lineages) in the USA. All but Delta and Omicron are categorized as Variants Being Monitored (VBM). It is reported that emerging variants, including B.1.1.7, B.1.351, P.1, and B.1.617.2 exhibit enhanced infectivity and viral production in vivo, which has also been confirmed in in vitro cell culture studies^56–58^. We observed similar trends in the ALI tissue cultures. While these models cannot replace critical small animal models for viral research, they may be an important tool for rapid investigation of new viral variants, and can be used as an accessible and physiologically relevant human-based platform on which to prioritize compound selection for animal testing and further pre-clinical evaluation. Also, these models can readily be deployed and utilized in laboratories that do not specialize in animal models, do not require pharmacokinetics studies to be carried out prior to antiviral evaluation, and can be miniaturized to 96 well format and scaled up to include dose-response testing. Indeed, in both the tracheobronchial and the alveolar ALI models, we were able to demonstrate robust antiviral activity of known anti-SARS-CoV-2 compound remdesivir, while also excluding hit compounds from 2D monolayer culture systems. Although we observed similar antiviral activity in both models, it is possible that host targeting antivirals may have different efficacy in the different tissue types. It is also worth noting that viral infection read-out in these tissues requires a whole tissue approach such as RNA quantification, viral titer, or staining and imaging of the entire tissue rather than select areas, due to cellular heterogeneity in the tissues, especially in the tracheobronchial tissues (i.e., patches of high alpha-tubulin regions rather than uniform throughout entire tissue). Whether ALI tissue models can provide more accurate results regarding their efficacy in vivo requires to be further evaluated and must be taken into consideration with a compound’s pharmacokinetic properties.

Several other ALI tissue models are described within the last year to assess both IAV and CoV infectivity, including SARS-CoV-2, each with advantages and limitations. Transwell-based URT and LRT ALI models have been shown to be a platform for antiviral drug discovery and support the replication of human coronaviruses (HCoV) with limited host cell range including SARS-CoV-2, SARS-CoV, HCoV-229E, HCoV-HKU1, HCoV-NL63, and HCoV-OC43^26–36,82–90^. Small airway and alveolar lung-on-chip models have been published or reported in pre-prints, although many of the lung-on-chip systems are currently low throughput, not readily compatible with laboratory automation used in drug screening facilities, and in some cases chips are made of polydimethylsiloxane PDMS, thus limiting its use for drug testing^31,83,84,91–93^. The use of a transwell-based multi-well plate assay platform as described in our study enables a versatile and modular approach for the future biofabrication of tissue ALI models with tailored physiological complexity and disease relevance. As an example, we have reported the use of bioprinting technique to create a vascularized skin tissue^94^ and the same approach can be applied to recreate a vascularized lung ALI tissue model. Addition of non-lymphocyte immune cells has also been explored using transwell plates with biofabricated tissue equivalents^95^ which can be applied to the lung ALI assay systems to assess the participation of innate immune cells in infectivity and COVID-19 relevant immune responses.

In summary, we have described the characterization of two distinct lower respiratory tract lung epithelial ALI tissue models for studying SARS-CoV-2 and IAV infection in relation to complex tissue-related disease. We established differential transcriptomic and inflammatory profiles induced by tracheobronchial *vs*. alveolar ALI tissues in response to two pandemic respiratory viruses, the recently emerged coronavirus SARS-CoV-2 and IAV, including a variant of the 2009 pH1N1. In addition, using known and novel antivirals we demonstrated the pharmacological validity of these two models as antiviral drug screening assay platforms. This characterization will serve as the foundation to bio-fabricate lung ALI tissue models, which may include additional physiological features that are relevant for the infection of respiratory viruses and the disease that they cause.

## Methods

### Viral propagation

Vero E6 cells were obtained from the American Type Culture Collection (ATCC CRL-1586) and cultured at 37 °C, 5% CO_2_ in DMEM with 10% fetal bovine serum (FBS), 1% penicillin/streptomycin, and 1% L-Glutamine. SARS-CoV-2 USA-WA1/2020 viral stocks were generated as previously described^12,57,96^. Briefly, Vero E6 cells were cultured in DMEM with 2% FBS + 10 mM HEPES buffer for 1 day prior to inoculation with the SARS-CoV-2 USA-WA1/2020 strain (BEI resources, NR-52281) (GenBank MN985325.1) using a low multiplicity of infection (MOI, 0.001), in order to generate an initial viral seed stock. At 72 hpi, tissue culture supernatants were collected and clarified by centrifugation, aliquoted and stored at −80 °C. The virus stock obtained from BEI Resources was a passage 4 (P4) stock and was used to generate a master seed stock (P5, or P0’) and working stock (P6, or P1’). Viral stock titers were determined by standard plaque forming assay (PFU/ml) as described below. Only stocks passaged once after seed stock (P1’) were used for experiments. SARS-CoV-2 USA-WA1/2020 used for comparison infection studies to SARS-CoV-2 variants B.1.1.7, B1.351, and Delta (B.1.617.2) were prepared by the SARS-CoV-2 Virology Core facility at NIAID. SARS-CoV-2 variant P.1. was obtained from BEI resources and expanded in Vero-TMPRSS2 cell lines as above. All work with infectious SARS-CoV-2 was carried out in a biosafety level 3 (BSL3) facility following approved protocols.

IAV H1N1 strains A/Puerto Rico/8/1934 (PR8) and A/California/07/2009 (pH1N1) were propagated in the allantoic cavity of 11 days-old embryonated chicken eggs. At 48 hpi, allantoic fluid were collected and aliquots stored at −80 °C.

### Culturing of human 3D in vitro respiratory tissue models

Human tracheobronchial air liquid interface (ALI) cultures (“Epiairway”) and human alveolar ALI cultures (“Epialveolar”) were obtained from MatTek Life Sciences (MA, USA) and cultured according to the manufacturer’s recommended protocol. Epiairway tissues were obtained at day 15 or day 21 post-seeding of primary donor lung cultures. Epiairway tissues obtained at day 15 post-seeding of primary donor lung cultures were further matured in-house in ALI interface for 7 days after receiving with 5 ml basolateral media changes every other day, with mucus washes (400 ml 1X PBS on apical side) every 3-4 days during maturation, prior to infection. Epialveolar tissues were obtained at day 21 post seeding of primary donor lung cultures and reconstituted overnight with 5 ml and 75 μl of Epialveolar media at the basal and apical sides of the tissue, respectively. Medium was changed after overnight recovery, prior to infection. Every other day, 5 ml basolateral media changes (and 75 μl apical media changes for Epialveolar tissues) were performed in both tissues for the duration of the experiments. For all SARS-CoV-2 infection kinetic studies, tissues were infected at day 23.

### Viral infection of lung tissue equivalents with SARS-CoV-2 or IAV

Tracheobronchial tissues were infected at day 21–30 of maturation to maximize matured ciliated cell populations at time of infection. Highest infection was observed when tissues were infected at day 27-28. Prior to viral inoculation, mucus was removed by washing twice the apical surface of tissues with 400 µl of TEER buffer (1X PBS with magnesium and calcium). Tissue inserts were inoculated with 1×10^5^ PFU of SARS-CoV-2 (for 36 h time-points and antiviral drug screening) or MOI of 0.1 and 1 of IAV (A/Puerto Rico/8/1934 or A/California/04/2009) or MOI of 0.1, 1, 3, or 10 of SARS-CoV-2 (2019-nCoV/USA-WA1/2020) for 1 h (IAV) or 1-4 h (SARS-CoV-2). Viral inoculum was removed and tissue inserts washed with PBS before continued culture. Inserts were cultured in ALI at 37 °C, 5% CO_2_ for 24–36 h for antiviral compound validation or 24, 48, 72, and 144 h for viral kinetics profiling, with basal media changes every other day. Alveolar tissues were infected between day 21-28 with either SARS-CoV-2 or IAV in the manner described above. Highest infection was observed when tissues were infected at day 27-28 for 1 h (Fig. 4b). Multiplicities of infection were calculated based on an average of 600,000 cells/tissue insert for alveolar tissues, and 900,000-1e6 cells/tissue insert for tracheobronchial tissues. No pre-infection apical washes were carried out for alveolar tissues. Basal media was replaced with fresh media every other day. In alveolar cultures, 75 μl of apical media was exchanged every other day. For both tracheobronchial and alveolar ALI tissues, mock-infected controls were treated in an identical fashion to viral inoculated controls.

### Plaque assay or TCID50 assay for SARS-CoV-2 production

At the corresponding time-points, secreted SARS-CoV-2 was captured by washing the apical tissue with 500 µl or 150 µl of pre-warmed tissue media (for tracheobronchial and alveolar ALI tissues, respectively). Plaque assay to determine viral loads of SARS-CoV-2-infected tissue culture supernatants was performed as previously described^57,96^.Briefly, Vero E6 monolayers in a 96-well plate format (4 × 10^4^ cells/well, performed in duplicate) were infected with 10-fold serial dilutions of collected apical supernatants in infection media (DMEM supplemented with 1% PSG). After viral adsorption (1 h at 37 °C, 5% CO_2_), cells were washed with PBS and incubated in post-infection media (DMEM supplemented with 2% FBS, 1% PSG) containing 1% microcrystalline cellulose (Avicel, Sigma-Aldrich) at 37 °C in 5% CO_2_ for 24 h. Plates were then inactivated in 10% neutral buffered formalin (ThermoFisher Scientific) for another 24 h prior to removal from the BSL3. For immunostaining, fixed monolayers were washed with PBS three times, permeabilized with 0.5% Triton X-100 for 15 min at room temperature (RT), and blocked with 2.5% bovine serum albumin (BSA in PBS) for 1 h at 37 °C, followed by incubation with a SARS-CoV N cross-reactive monoclonal antibody (MAb, at 1 µg/ml), 1C7C7, diluted in 1% BSA for 1 h at 37 °C. After incubation with the primary MAb, cells were washed three times with PBS, and developed with the Vectastain ABC kit and DAB Peroxidase Substrate kit (Vector Laboratory, Inc., CA, USA) according to the manufacturers’ instructions. Viral counts were performed using the C.T.L. Immunospot v7.0.15.0 Professional Analysis DC and calculated as PFUs/tissue. For TCID50 assay, Vero-TMPRSS2 (BPS Bioscience) monolayers in a 96-well plate format (4 × 104 cells/well, performed in duplicate) were infected with 10-fold serial dilutions of collected apical supernatants in infection media (DMEM supplemented with 2% FBS) and incubated for 72 hours at 37 °C, 5% CO_2_. Wells were then fixed with 4% PFA and stained with 1% crystal violet. TCID50 units were calculated using the Reed–Muench method.

### Focus forming unit (FFU) assay for IAV

At indicated time-points, secreted IAV was captured by washing the apical side of the tissues with 200 µl of 1X PBS. IAV titers produced from tracheobronchial and alveolar ALI tissues were measured by focus forming unit assay. Rhesus monkey kidney epithelial cells LLC-MMK2, overexpressing SIAT1 were seeded 1 day prior in black, 96-well, clear bottom plate to reach a confluency of 95-100% at time of FFU assay. Apical washes containing secreted virus from lung tissue equivalents was diluted in 2% FBS containing EMEM media and used to inoculate LLC-MMK2-SIAT1 cells for 2 h at 37 °C. Viral inoculum was removed and replaced with an Avicel-media overlay and cells incubated at 37 C/5%CO2 for 48 h. After 48 h, the overlay was removed, cells washed twice with 1X PBS prior to fixation with 4% paraformaldehyde. Fixed cells were washed with 1X PBS three times prior to immunostaining for IAV NP protein and counterstain with Hoechst. All plates were imaged on the InCell2200 and FFU quantified using Columbus Analysis software. All antibodies can be found in Supplementary Table 2.

### Drug treatments

All compounds were dissolved in DMSO unless otherwise specified. DMSO or compounds were diluted at indicated concentration directly into the basolateral media chamber of the tissue inserts for one hour prior to viral exposure and remained in the media for duration of experiment (24 h for IAV, 36 h to 72 h for SARS-CoV-2). Hydroxychloroquine was dissolved in water.

### Immunofluorescence staining and analysis

Tissue inserts were inactivated according to institute SOP. Tissue inserts were completely submerged in 4% paraformaldehyde (PFA) solution for a minimum of 1.5 h or 30 min (if analyzed inside the BSL3) in 12- or 24-well plates (for EpiAlveolar and Epiairway tissues, respectively) before removal of PFA and washing with PBS three times. SARS-CoV-2 tissue inserts processed at the NIH were completely submerged in 10% NBF for a minimum of 72 hours to inactivate virus. Tissues were permeabilized in a 0.3–0.5% Triton X-100 in PBS solution for 15 min, followed by blocking in PBTG (1% BSA + 5% goat serum + 0.1% Triton X-100 in PBS) for 30 min to 1 h at RT or overnight at 4 °C. Tissues were then stained directly in inserts or removed from inserts using a scalpel and stained whole or as cut into four equal quarters. Primary antibodies were diluted in PBTG (see Supplementary Table 2) and incubated at 4 °C overnight or for 1 h at 37 °C. Secondary antibodies were also diluted in PBTG (1% BSA + 5% goat serum + 0.1% Triton X-100 in PBS) and incubated for 2 h at RT or 1 h at 37 °C followed by three washes with 1X PBS. Hoechst or DAPI were used to stain DNA (nuclei) of tissues infected with IAV and SARS-CoV-2, respectively. Tissues were imaged in the transwell insert or mounted in glass-bottom plates in an automated high content confocal microscope (Opera Phenix, Perkin Elmer) or using the Cytation 5 cell imaging multi-mode reader (Biotek) at ×4 magnification, WFOV mode with laser autofocus; whole well images were acquired and analyzed using Gen 5 v3.8.01 software.

### qRT-PCR for quantification of SARS-CoV-2 RNA

Total RNA was isolated using TRIzol Reagent (Invitrogen) and purified using RNA Clean & Concentrator Kits (Zymo Research). 1 µg of total RNA was used to synthesize cDNA using the M-MLV Reverse Transcriptase Kit with Random Primers (Invitrogen). Gene specific primers targeting 18 S RNA (forward:AACCCGTTGAACCCCATT, reverse:CCATCCAATCGGTAGTAGCG) or the SARS-CoV-2 N gene (forward: TTACAAACATTGGCCGCAAA, reverse: GCGCGACATTCCGAAGAA) and Power SYBR Green PCR Master Mix (Applied Biosystems) were used to amplify cellular RNA and viral RNA by QuantStudio 6 Flex Real-Time PCR Systems (Applied Biosystems). The relative expression levels of SARS-CoV-2 N gene was calculated using the standard curve method and normalized to 18 S ribosomal RNA as an internal control.

### Tissue dissociation and scRNAseq and data processing

Tissues were infected with 1 × 10^5^ TCID_50_ units PR8 H1N1 IAV (A/Puerto Rico/8/1934) for 48 h or of 1xe10^6^ TCID_50_ SARS-CoV-2 (2019-nCoV/USA-WA1/2020) for 72 hours. Tissues were dissociated by submerging the tissues into 1X PBS with calcium and magnesium for five minutes (apical side of tracheobronchial ALI tissues was previously washed twice with 1x PBS to remove mucus), followed by 5 minutes incubation in 0.1 M EDTA, followed by incubation in 0.25% trypsin for 12 minutes (alveolar ALI tissues) or -20 (tracheobronchial ALI tissues) minutes and pipetted up and down to dissociate the tissue. Cells were then carefully collected in ice cold EMEM media (ATCC), filtered first through a 100 ${{{{{\rm{\mu}}}}}}$M strainer to remove cell aggregates, centrifuged at 300 x *g* for 5 min at 4 °C and resuspended in 1 mL of 1X PBS + 0.04% BSA, and finally filtered with 70 ${{{{{\rm{\mu}}}}}}$M Flowmi filters (Sigma). The cell concentration for each sample was determined using a Countess II automatic cell counter. Single cell suspensions were then processed using the 10X Genomics Platform according to manufacturer instructions with v3 reagent and sequenced on the Illumina NexSeq550 Platform (Illumina). The raw data were demultiplexed and mapped to human reference genome with virus genomes (PR8-IAV, segments 1–8: GenBank accession: NC_002023.1, NC_002021.1, NC_002022.1, NC_002017.1, NC_002019.1, NC_002018.1, NC_002016.1, NC_002020.1; WA1-SARS-CoV2, GenBank accession: MN985325 Version) using CellRanger (10x Genomics) with standard default pipeline parameters. Raw count matrix for each sample was imported into an R pipeline using Seurat v4 package^97^. Low quality cells (<200 genes, <400 UMI, < 0.8 gene complexity (log10GenesPerUMI) and >0.2 mitochondrial ratio) were filtered out from analysis. Additionally, genes which were expressed in less than 10 cells were excluded from downstream analysis. Doublet cells were further removed by running DoubletFinder R package^98^. Data from each tissue were then normalized, scaled and log-transformed with Seurat packages using the SCTransform method^97^.

### Cell type identification

The cell types were identified by differential gene expression (DEG) between clusters using Seurat FindAllMarkers and further annotated by using cell-type-specific single-cell signatures from respective cell atlases and curated publications^99^. Labels were added to the main object as cell-type identities.

### Differential analysis

For each tissue model, Differential Gene Expression (DEG) Analyses were applied by comparing “tissue type”, “type of virus”, “Infection status” with Seurat FindAllMarkers, and FindMarkers functions^97^. The visualization plots were generated using R packages (EnhancedVolcano and ggplot2)^100–103^. The lists of DEGs were saved for further enrichment analysis. The significance cut-off for EnrichR enrichment analysis was Log_2_FC = 0.264 (~1.2 fold change), and Log_2_FC = 0.7 for volcano plots with adjusted *p*-values shown.

### Enrichment pathway analysis

The enrichment analysis was performed using R package EnrichR (libraries:BioPlanet_2019,KEGG_2021_Human,WikiPathways_2021_Human, MSigDB_Hallmark_2020, GO_Biological_Process_2021, GO_Molecular_Function_2021), fgsea (library c5.ontology gene sets all.v7.4) and clusterprofiler (library Go.db 2.1) packages with selected DEG list from the differential analysis (described above). The combined expression of the genes in each enrichment category was calculated using AddModuleScore function in Seurat package^101–111^.

### Visualization

All plots were generated using Seurat visualization functions, ggplot2, Complexheatmap, and EnhancedVolcano R packages^97,100–102^. Venn diagrams were made with (http://bioinformatics.psb.ugent.be/webtools/Venn/).

### Cytokine and chemokine quantification

Basal media was collected at different time-points (24, 48, 72, and 144 hpi) from both tracheobronchial and alveolar infected ALI tissues and used to measure TH1/TH2 responses and growth factors with a customized 22-multiplex panel Human Magnetic bead Luminex assay (R&D Systems, MN, USA), following the manufacturer’s instructions. Luminex assays were performed in the BSL3 and final samples decontaminated by an overnight incubation in 1% formalin solution before readout on a Luminex 100/200 System running on Xponent v4.2, with the following parameters: gate 8000–16,500, 50 μl of sample volume, 50–100 events per bead, sample timeout 60 s, low PMT (LMX100/200: Default). Acquired data were analyzed using Millipore Sigma Belysa™ v1.0.

### Statistics and reproducibility

Statistical significance was determined using Prism v9.0.1 software (GraphPad Software, San Diego, CA). The unpaired, two-tailed Student’s *t* test was used for two group comparisons for each time-point and reported as *p  <  0.05; **p  <  0.005; ***p  <  0.0005, ****p  <  0.00005.

### Reporting summary

Further information on research design is available in the Nature Research Reporting Summary linked to this article.

## Supplementary information

Supplementary Information

Description of Additional Supplementary Files

Supplementary Data 1

Supplementary Data 2

Supplementary Data 3

Reporting Summary

## Data Availability

The datasets generated during and/or analysed during the current study are available from the corresponding authors on reasonable request. Raw data for graphed experiments can be found in Supplementary Data 3).
