# Supplementary material for: Modeling SARS-CoV-2 and influenza infections and antiviral treatments in human lung epithelial tissue equivalents
Source: Commun Biol. 2022 Aug 12;5:810. doi: 10.1038/s42003-022-03753-7 (PMC9373898; doi:10.1038/s42003-022-03753-7)
Supplement: Supplementary file 1 — Supplementary Information [file 42003_2022_3753_MOESM1_ESM.pdf]

Supplemental Figures

Modeling SARS-CoV-2 and Influenza Infections and Antiviral Treatments in Human Lung Epithelial Tissue Equivalents

Running title: 3D Lung Tissue equivalents for SARS-CoV-2 Infection and Antiviral Discovery

Hoda Zarkoob[1,11], Anna Allué-Guardia[2,11], Yu-Chi Chen[1], Andreu Garcia-Vilanova[2], Olive Jung[1,3], Steven Coon[4], Min Jae Song[1], Jun-Gyu Park[2], Fatai Oladunni[2], Jesse Miller[5,6,7], Yen-Ting Tung[1], Ivan Kosik[8], David Schultz[5,9], James Iben[4], Tianwei Li[4], Jiaqi Fu[1], Forbes D. Porter[10], Jonathan Yewdell[8], Luis Martinez-Sobrido[2], Sara Cherry[5,6,7], Jordi B. Torrelles[2], Marc Ferrer[*1], Emily M. Lee[*1]

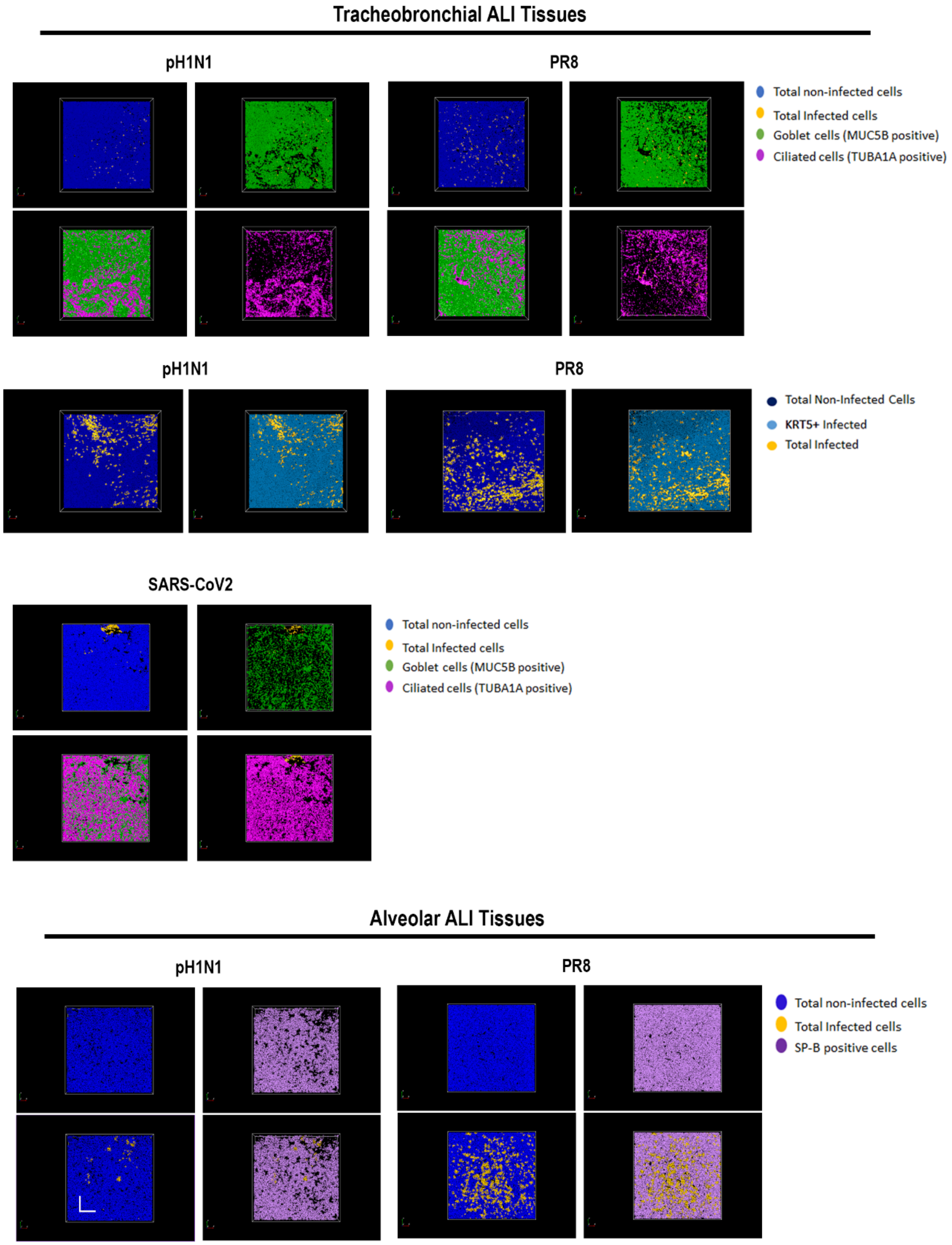

Tracheobronchial ALI Tissues
pH1N1
PR8
Total non-infected cells
Total Infected cells
Goblet cells (MUC5B positive)
Ciliated cells (TUBA1A positive)
pH1N1
PR8
Total Non-Infected Cells
KRT5+ Infected
Total Infected
SARS-CoV2
Total non-infected cells
Total Infected cells
Goblet cells (MUC5B positive)
Ciliated cells (TUBA1A positive)
Alveolar ALI Tissues
pH1N1
PR8
Total non-infected cells
Total Infected cells
SP-B positive cells

Supplemental Figure 1: Biorender image of co-staining of cellular markers and viral antigen. Tracheobronchial and alveolar ALI tissues were infected with IAV pH1N1 or PR8 at MOI of ~0.1, and SARS-CoV-2 at MOI of ~0.1 and fixed at 24hpi (IAV) or 36hpi (SARS-CoV-2). Representative stained images of tracheobronchial ALI tissues with Hoechst (nuclei marker, blue), α -tubulin (ciliated cell marker, magenta), MUC5B (goblet cell marker, green) and KRT5 (basal cell marker, aqua). In orange is anti-IAV NP or anti-SARS-CoV-2 S/N proteins. Bottom, alveolar: Representative stained images of alveolar ALI tissues with Hoechst (nuclei marker, blue), surfactant protein B (SP-B, ATII/pneumonocyte type II cell marker, purple) and anti-IAV NP or anti-SARS-CoV-2 spike/nucleocapsid (orange). Biorender images were created using Columbus High Content Profiler (Perkin Elmer). Scale bar is 200 µm.

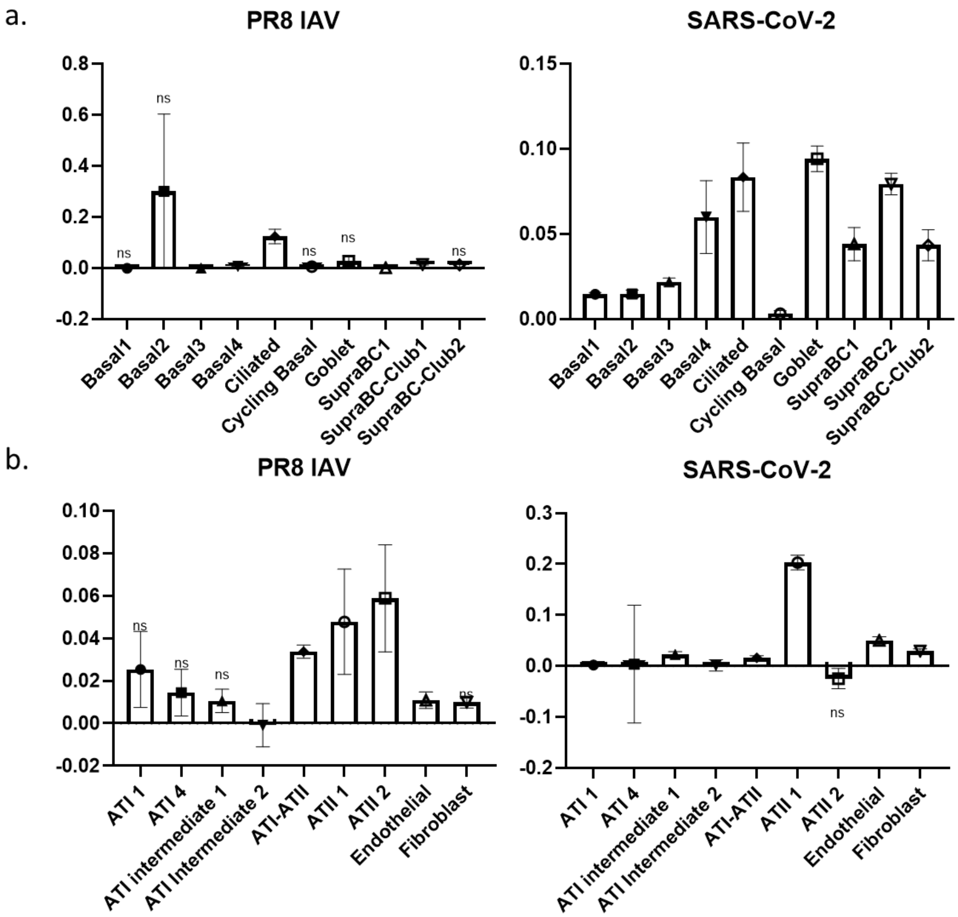

Supplemental Figure 2: Module scoring of ScRNAseq of IAV or SARS-CoV-2 infected lung ALI tissues. Tracheobronchial and alveolar ALI tissues were infected with PR8 IAV (1e5 TCID50 units/tissue, n=2) for 48 h or WA1-SARS-CoV-2 (2e5 PFU/tissue, n=2) for 72 h prior to dissociation for scRNAseq. Shown here are module scorings of **a)** tracheobronchial ALI tissues infected with PR8 IAV for 48 h (left), or with SARS-CoV-2 for 72 h (right) and **b)** alveolar ALI tissues infected with PR8 IAV for 48 h (left), or with SARS-CoV-2 for 72 h. Module scoring was calculated by using the average expression level of SARS-CoV-2 or IAV viral gene clusters on the single cell level, subtracted by the aggregated expression of randomly selected control feature sets (host genes). All values are statistically significant with p values <0.05 unless noted as not significant (ns). The statistics are t-test (two-sided, Welch Two Sample t-test, confidence level =0.95)

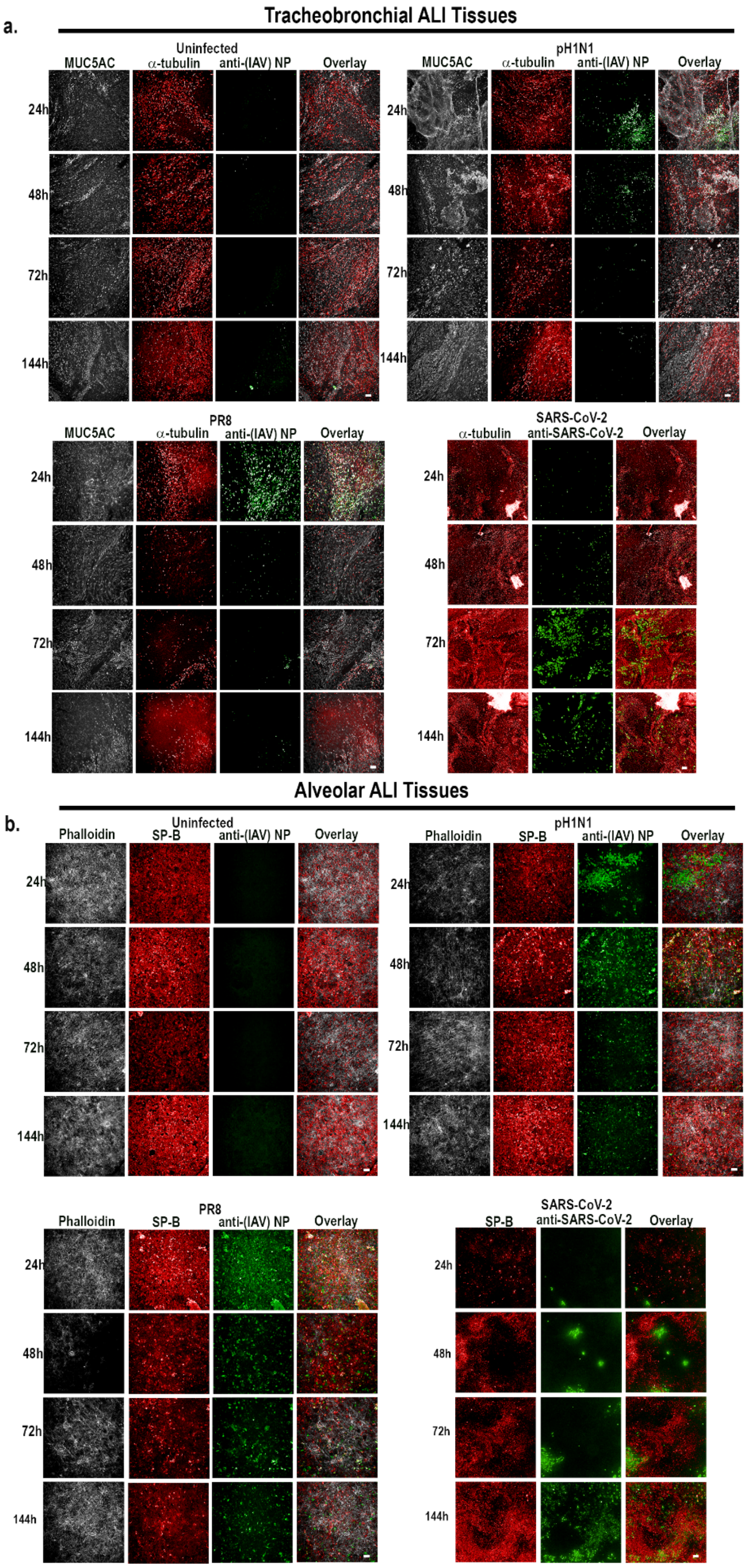

a.
Tracheobronchial ALI Tissues
Uninfected
MUC5AC
α-tubulin
anti-(IAV) NP
Overlay
pH1N1
24h
48h
72h
144h
PR8
SARS-CoV-2
anti-SARS-CoV-2
Alveolar ALI Tissues
b.
Phalloidin
SP-B

Supplemental Figure 3: Co-staining of additional cellular markers from infection time course in Figure 3. Tracheobronchial and alveolar ALI tissues were infected with IAV pH1N1 or PR8 at MOI of 0.1, and SARS-CoV-2 at MOI of 1 (fixed tissue samples shown) or as indicated in titer plots. Apical washes were collected and tissues fixed at 24, 48, 72 and 144 hpi. **a)** Tracheobronchial and **b)** alveolar ALI tissues were stained with anti-IAV N protein and anti-SARS-CoV-2 N/spikeS/N proteins monoclonal antibody cocktail to label infected cells (shown in green) as well as **a)** MUC5AC (white), alpha-tubulin (red) or **b)** phalloidin (white), and surfactant B protein (red). Scale bar is 100 µm and 200 µm in IAV and SARS-CoV-2 infected tissues, respectively.

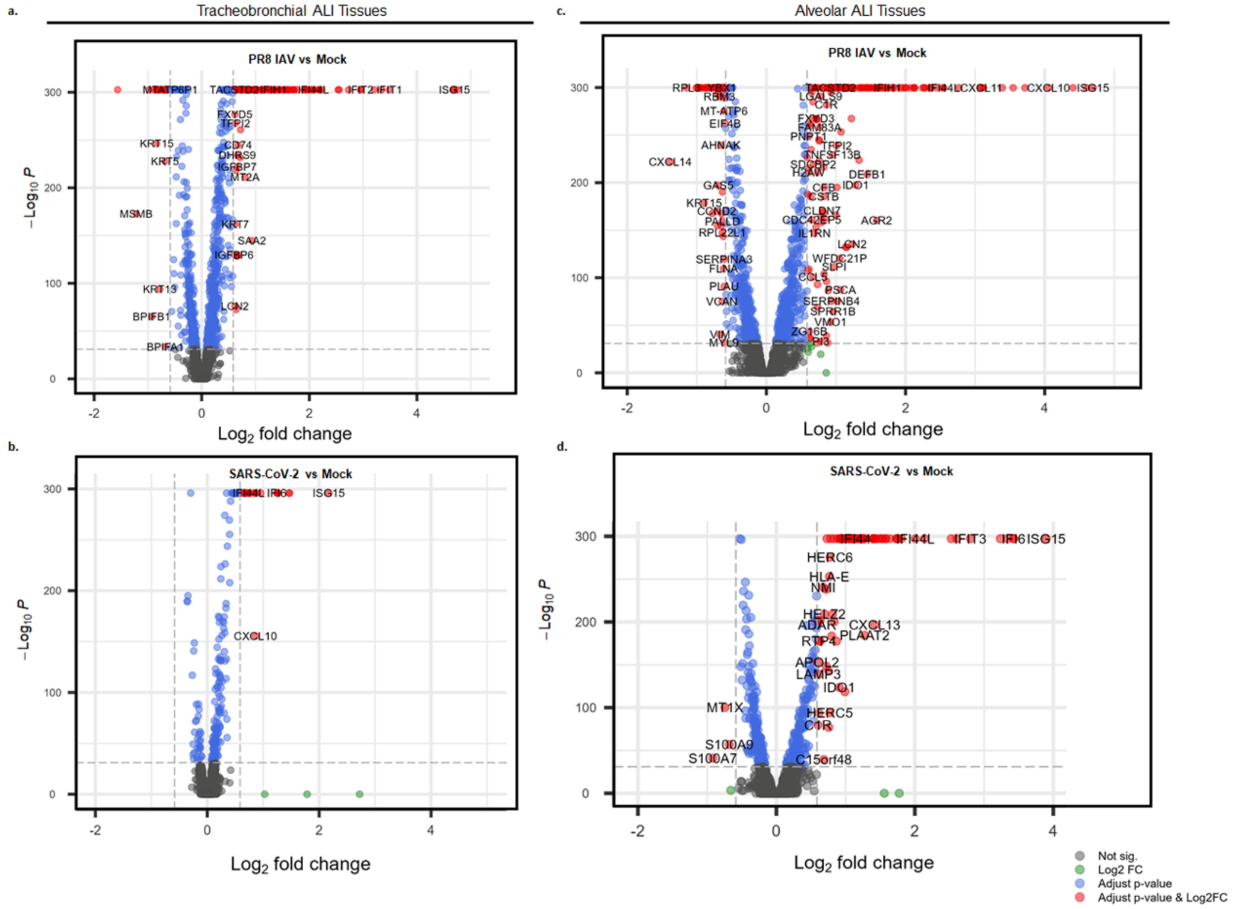

Supplemental Figure 4. Volcano plot of major gene expression shifts ($\log_2$ FC) in tracheobronchial mock infected tissues *vs.* **a)** 48 hpi PR8-IAV or **b)** 72 hpi SARS-CoV-2 infected tracheobronchial

tissues, or alveolar mock-infected tissues *vs*. **c)** 48 hpi PR8-IAV or **d)** 72 hpi SARS-CoV-2 infected alveolar tissues. In red, DEGs with log_2fc of 0.7 and adjusted p-value of <0.05.

a. Tracheobronchial ALI Tissue

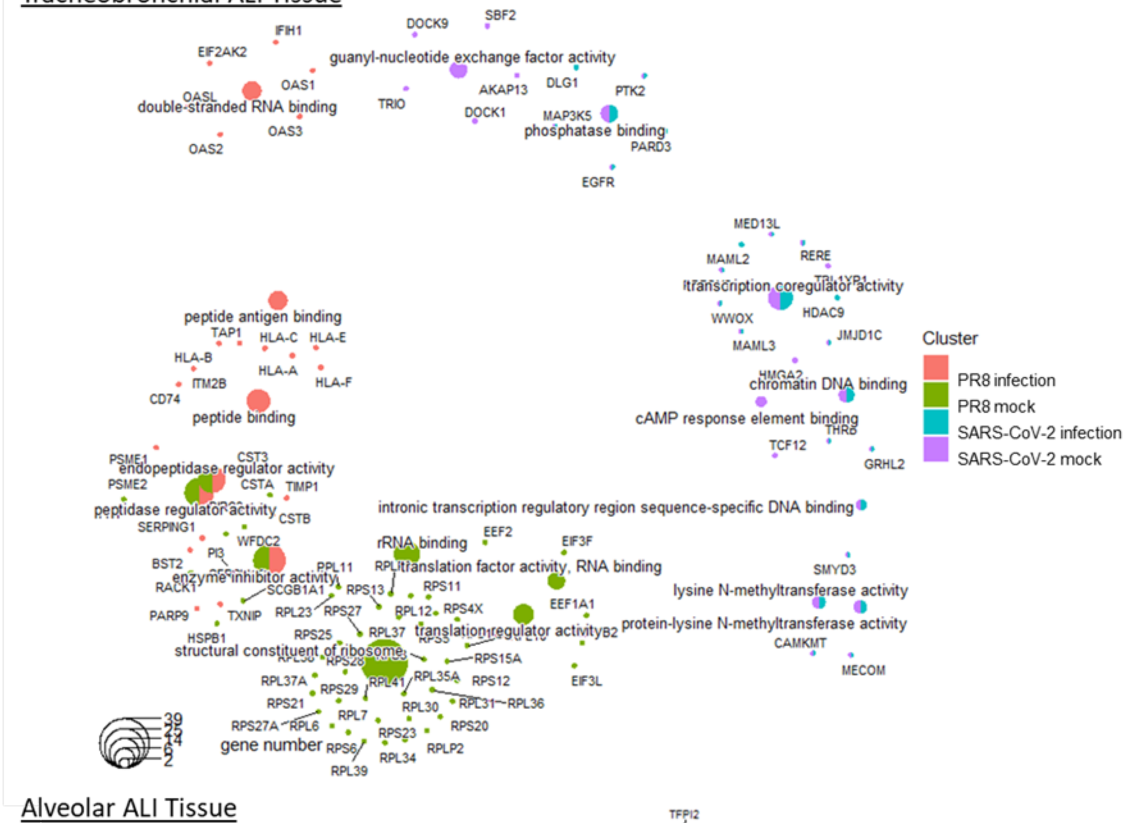

b. Alveolar ALI Tissue

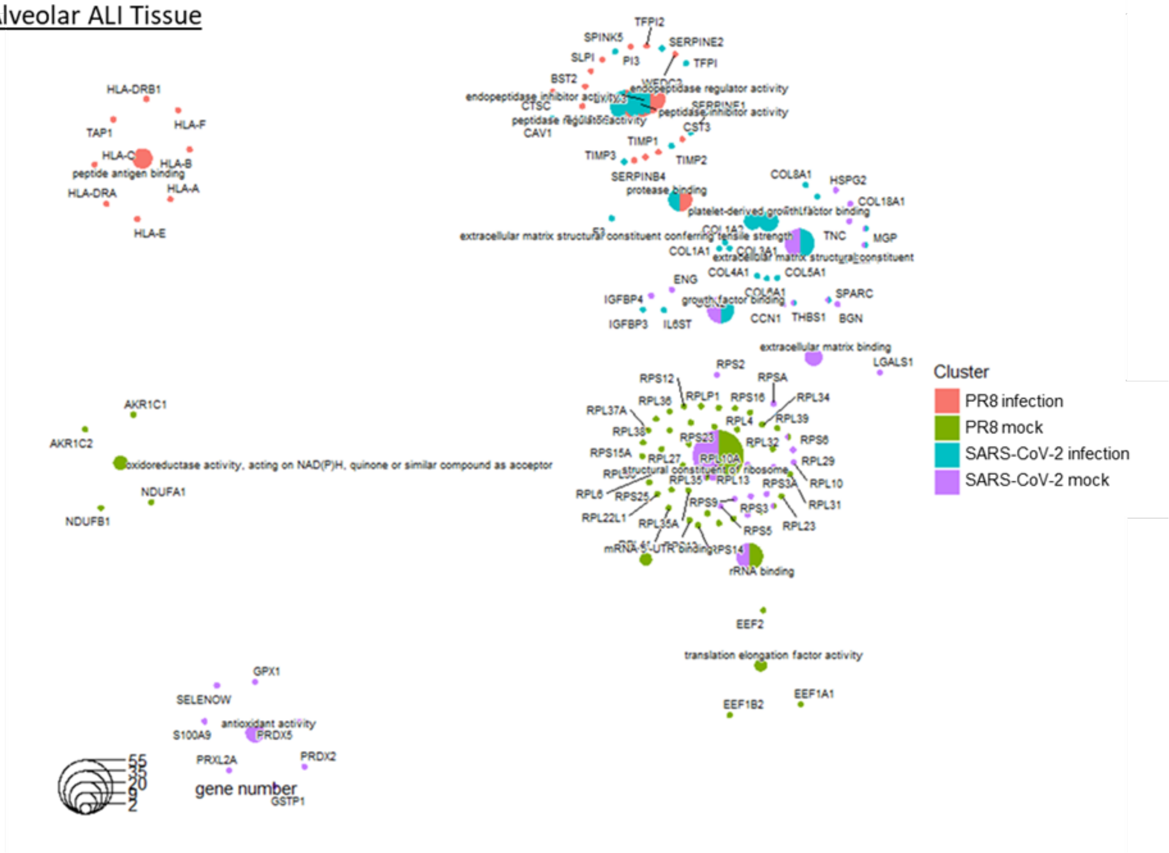

Supplemental Figure 5. a) Cluster map analysis of major divergent gene clusters between mock or infected PR8 IAV or SARS-CoV-2 tracheobronchial or **b)** alveolar ALI tissues. The size of the circle corresponds to the number of genes.

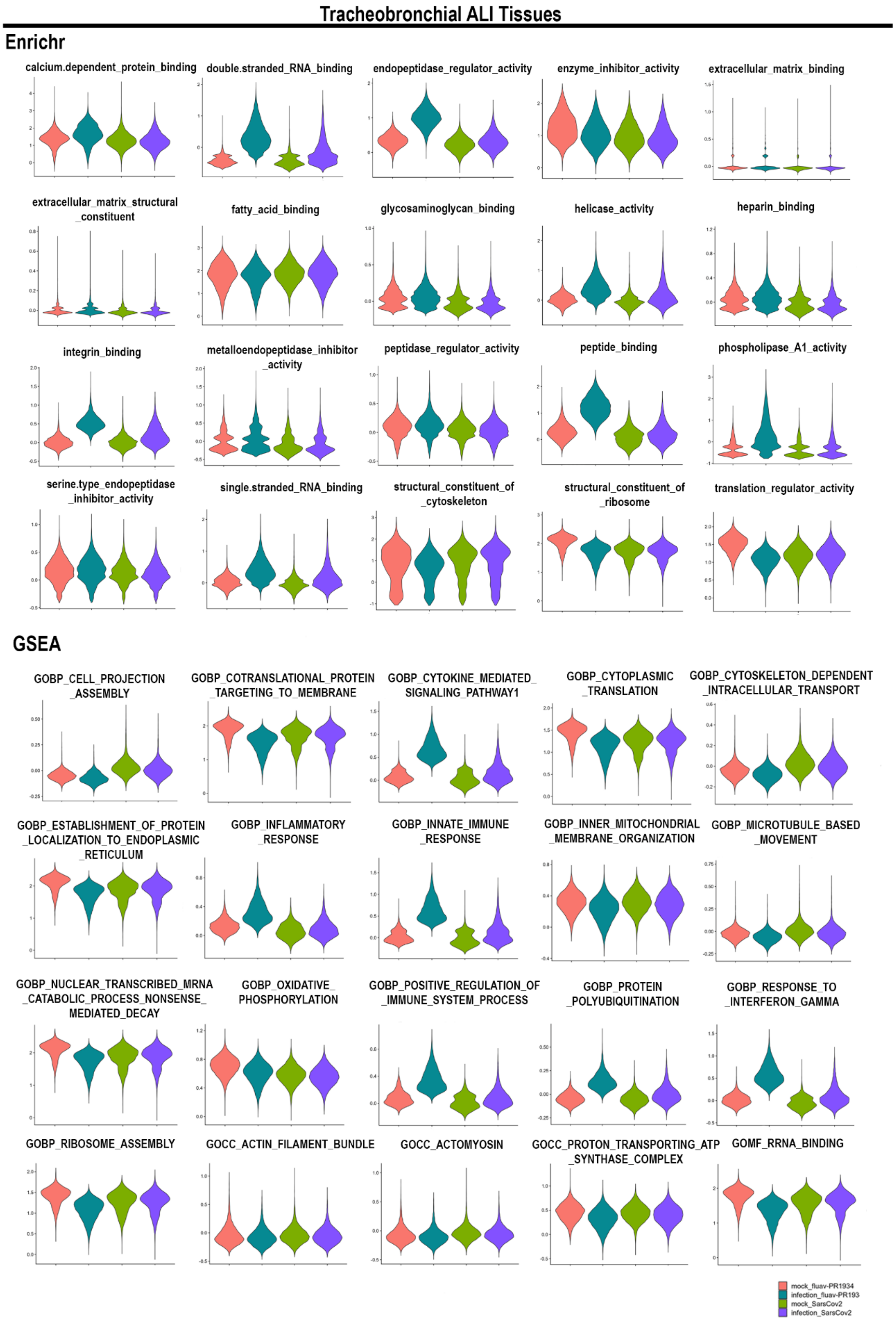
Tracheobronchial ALI Tissues
Enrichr
calcium.dependent_protein_binding
double.stranded_RNA_binding
endopeptidase_regulator_activity
enzyme_inhibitor_activity
extracellular_matrix_binding
extracellular_matrix_structural_constituent
fatty_acid_binding
glycosaminoglycan_binding
helicase_activity
heparin_binding
integrin_binding
metalloendopeptidase_inhibitor_activity
peptidase_regulator_activity
peptide_binding
phospholipase_A1_activity
serine.type_endopeptidase_inhibitor_activity
single.stranded_RNA_binding
structural_constituent_of_cytoskeleton
structural_constituent_of_ribosome
translation_regulator_activity
GSEA
GOBP_CELL_PROJECTION_ASSEMBLY
GOBP_COTRANSLATIONAL_PROTEIN_TARGETING_TO_MEMBRANE
GOBP_CYTOKINE_MEDIATED_SIGNALING_PATHWAY1
GOBP_CYTOPLASMIC_TRANSLATION
GOBP_CYTOSKELETON_DEPENDENT_INTRACELLULAR_TRANSPORT
GOBP_ESTABLISHMENT_OF_PROTEIN_LOCALIZATION_TO_ENDOPLASMIC_RETICULUM
GOBP_INFLAMMATORY_RESPONSE
GOBP_INNATE_IMMUNE_RESPONSE
GOBP_INNER_MITOCHONDRIAL_MEMBRANE_ORGANIZATION
GOBP_MICROTUBULE_BASED_MOVEMENT
GOBP_NUCLEAR_TRANSCRIBED_MRNA_CATABOLIC_PROCESS_NONSENSE_MEDIATED_DECAY
GOBP_OXIDATIVE_PHOSPHORYLATION
GOBP_POSITIVE_REGULATION_OF_IMMUNE_SYSTEM_PROCESS
GOBP_PROTEIN_POLYUBIQUITINATION
GOBP_RESPONSE_TO_INTERFERON_GAMMA
GOBP_RIBOSOME_ASSEMBLY
GOCC_ACTIN_FILAMENT_BUNDLE
GOCC_ACTOMYOSIN
GOCC_PROTON_TRANSPORTING_ATP_SYNTHASE_COMPLEX
GOMF_RRNA_BINDING
mock_fluav-PR1934
infection_fluav-PR193
mock_SarsCov2
infection_SarsCov2

Supplementary Figure 6: Pathway enrichments per tracheobronchial ALI tissue per virus. Violin plots of module scoring from select **a)** Enrichr or **b)** GSEA pathway analysis of uninfected, PR8-IAV-infected, or SARS-CoV-2 infected tracheobronchial ALI tissues.

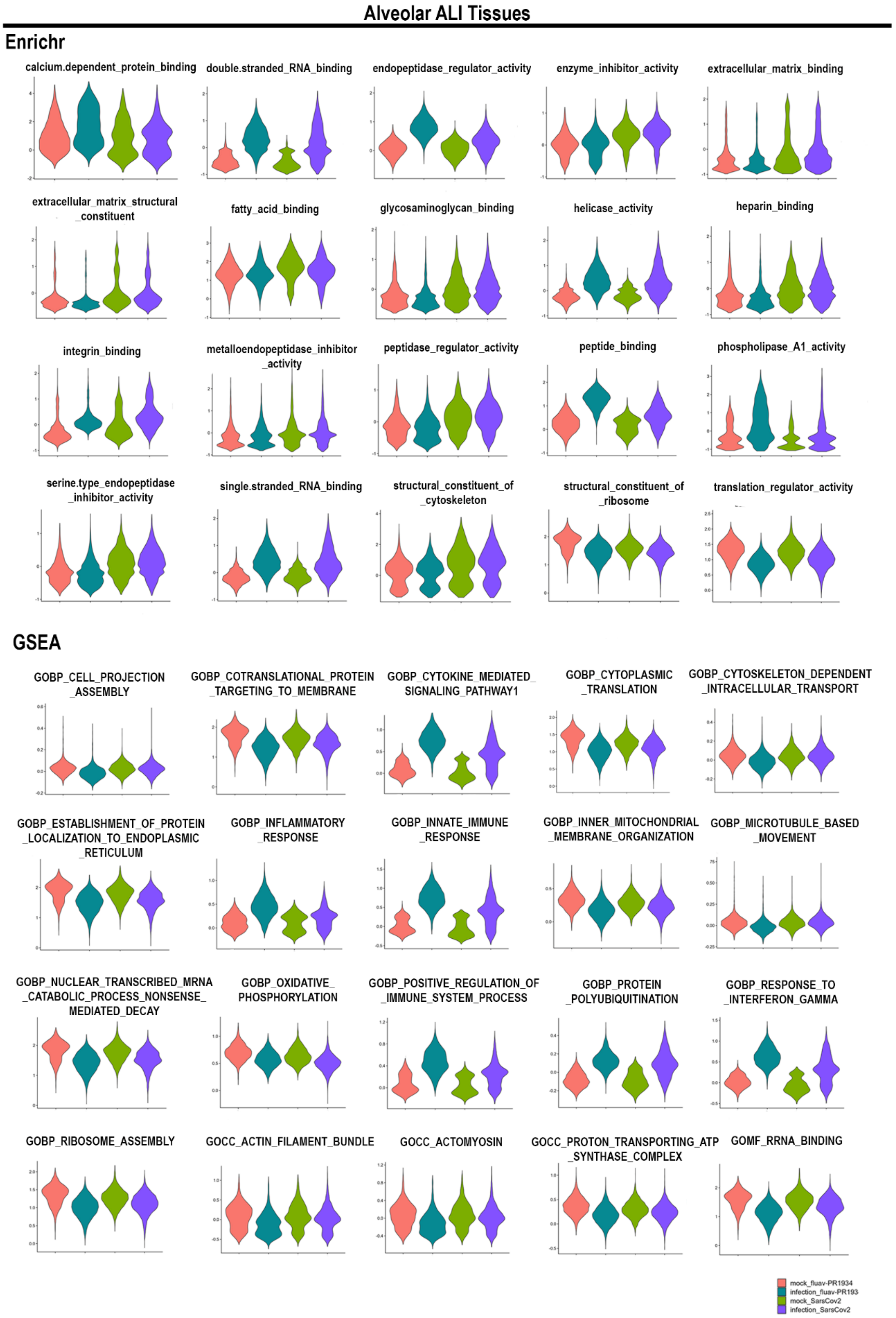

Alveolar ALI Tissues
Enrichr
calcium.dependent_protein_binding
double.stranded_RNA_binding
endopeptidase_regulator_activity
enzyme_inhibitor_activity
extracellular_matrix_binding
extracellular_matrix_structural_constituent
fatty_acid_binding
glycosaminoglycan_binding
helicase_activity
heparin_binding
integrin_binding
metalloendopeptidase_inhibitor_activity
peptidase_regulator_activity
peptide_binding
phospholipase_A1_activity
serine.type_endopeptidase_inhibitor_activity
single.stranded_RNA_binding
structural_constituent_of_cytoskeleton
structural_constituent_of_ribosome
translation_regulator_activity
GSEA
GOBP_CELL_PROJECTION_ASSEMBLY
GOBP_COTRANSLATIONAL_PROTEIN_TARGETING_TO_MEMBRANE
GOBP_CYTOKINE_MEDIATED_SIGNALING_PATHWAY1
GOBP_CYTOPLASMIC_TRANSLATION
GOBP_CYTOSKELETON_DEPENDENT_INTRACELLULAR_TRANSPORT
GOBP_ESTABLISHMENT_OF_PROTEIN_LOCALIZATION_TO_ENDOPLASMIC_RETICULUM
GOBP_INFLAMMATORY_RESPONSE
GOBP_INNATE_IMMUNE_RESPONSE
GOBP_INNER_MITOCHONDRIAL_MEMBRANE_ORGANIZATION
GOBP_MICROTUBULE_BASED_MOVEMENT
GOBP_NUCLEAR_TRANSCRIBED_MRNA_CATABOLIC_PROCESS_NONSENSE_MEDIATED_DECAY
GOBP_OXIDATIVE_PHOSPHORYLATION
GOBP_POSITIVE_REGULATION_OF_IMMUNE_SYSTEM_PROCESS
GOBP_PROTEIN_POLYUBIQUITINATION
GOBP_RESPONSE_TO_INTERFERON_GAMMA
GOBP_RIBOSOME_ASSEMBLY
GOCC_ACTIN_FILAMENT_BUNDLE
GOCC_ACTOMYOSIN
GOCC_PROTON_TRANSPORTING_ATP_SYNTHASE_COMPLEX
GOMF_RRNA_BINDING
mock_fluav-PR1934
infection_fluav-PR193
mock_SarsCov2
infection_SarsCov2

Supplementary Figure 7: **Pathway enrichments per alveolar ALI tissue per virus**. Violin plots of module scoring from select **a)** Enrichr or **b)** GSEA pathway analysis of uninfected, PR8-IAV-infected, or SARS-CoV-2 infected alveolar ALI tissues.

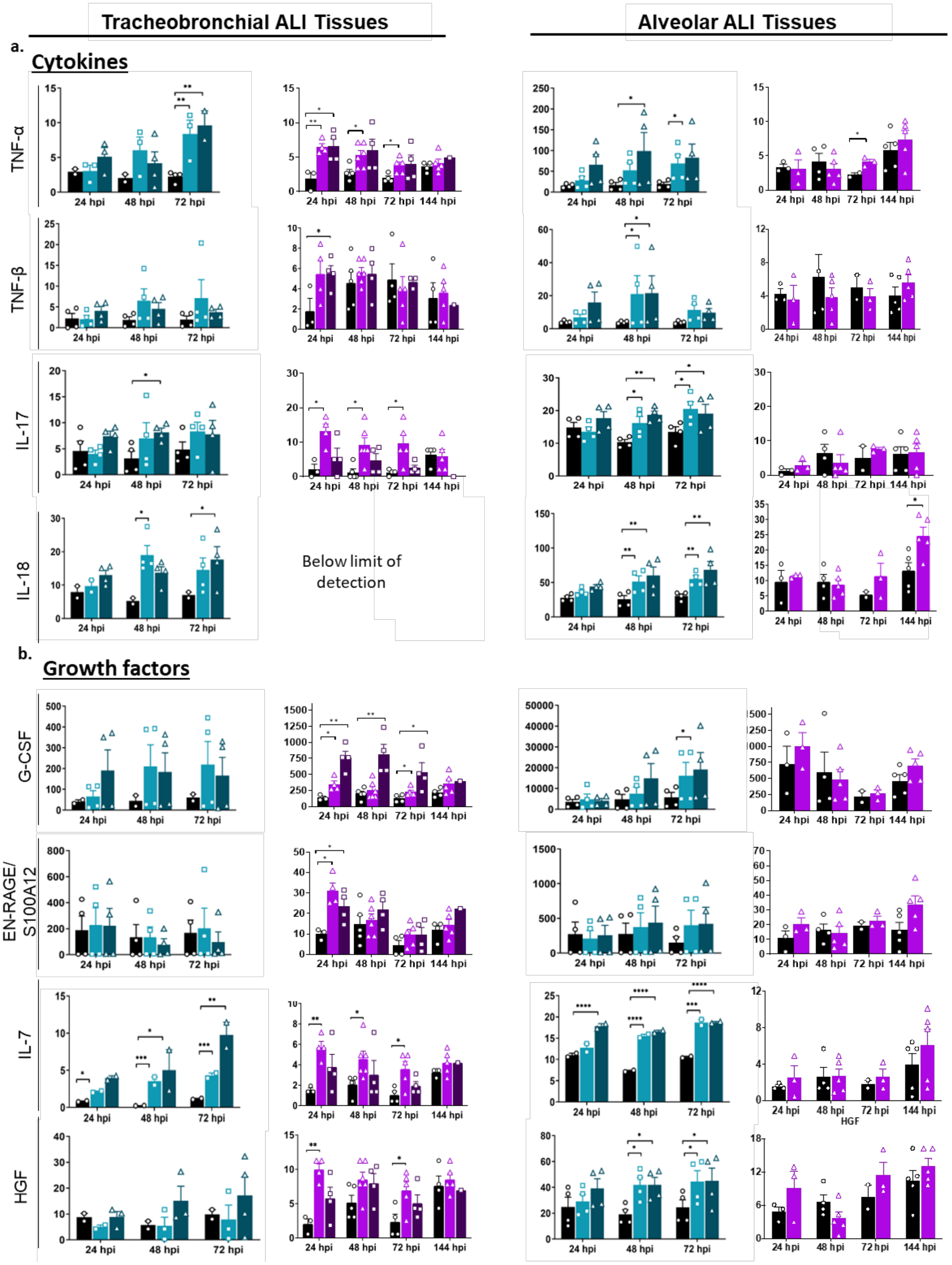

Tracheobronchial ALI Tissues
Alveolar ALI Tissues
a.
Cytokines
TNF-α
TNF-β
IL-17
IL-18
Below limit of detection
b.
Growth factors
G-CSF
EN-RAGE/ S100A12
IL-7
HGF
24 hpi
48 hpi
72 hpi
144 hpi

Supplemental Figure 8: Production of other inflammatory markers. Basal compartment media were collected from tracheobronchial (left two panels) or alveolar (right two panels) ALI tissues at indicated time-points and analyzed for cytokine and chemokine secretion by Luminex assay. IAV infected tissues (MOI of 0.1) are represented in shades of teal, where light teal shows infection with the IAV pH1N1 strain and dark teal shows infection with the IAV PR8 strain. SARS-CoV-2 infected tissues are represented in shades of purple, with progressing color from low MOI (~1) to high MOI (~10): **a)** Cytokines (TNF-α, TNF-β, IL-17, IL-18) and **b)** Growth factors (G-CSF, EN-RAGE/S100A12, IL-7, HGF). All measurements on y axis are in pg/ml. Data are represented as M±SEM for a minimum of n=3 independent experiments and/or biological replicates except for tracheobronchial MOI 10 at 144hpi n=1, and alveolar mock 72hpi (alveolar) n=2. Student t-test of IAV or SARS-CoV-2 infected tissues *vs*. uninfected controls at each timepoint: *$p < 0.05$, **$p < 0.005$, ***$p < 0.0005$, ****$p < 0.00005$.

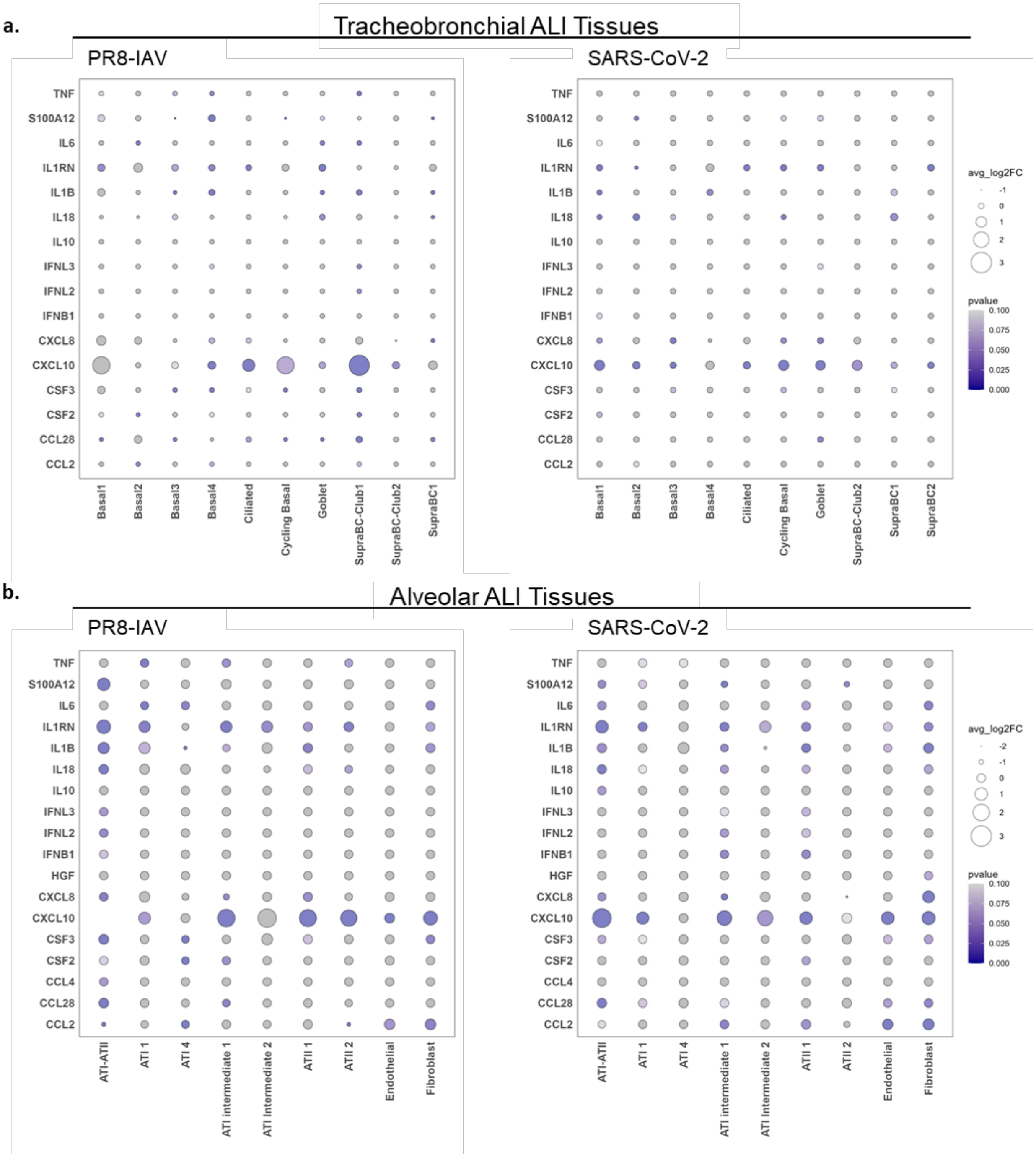

Supplemental Figure 9. Expression of select inflammatory related genes in cell types identified by scRNAseq after viral infection. Tracheobronchial and alveolar ALI tissues were infected with PR8 IAV (1e5 TCID50 units/tissue, n=2) for 48 h or WA1-SARS-CoV-2 (2e5 PFU/tissue, n=2) for 72 h prior to dissociation for scRNAseq. ScRNAseq was used to analyze 48 hpi PR8-IAV or 72 hpi SARS-CoV-2 samples in comparison to a custom Luminex panel of cytokines and chemokines. Dot

plot of relative mean expression of detected genes of interest related to the custom inflammatory Luminex panel in identified cell types in **a)** tracheobronchial tissues or **b)** alveolar tissues in uninfected, 48 hpi PR8-IAV infected, or 72 hpi SARS-CoV-2 infected tissues.

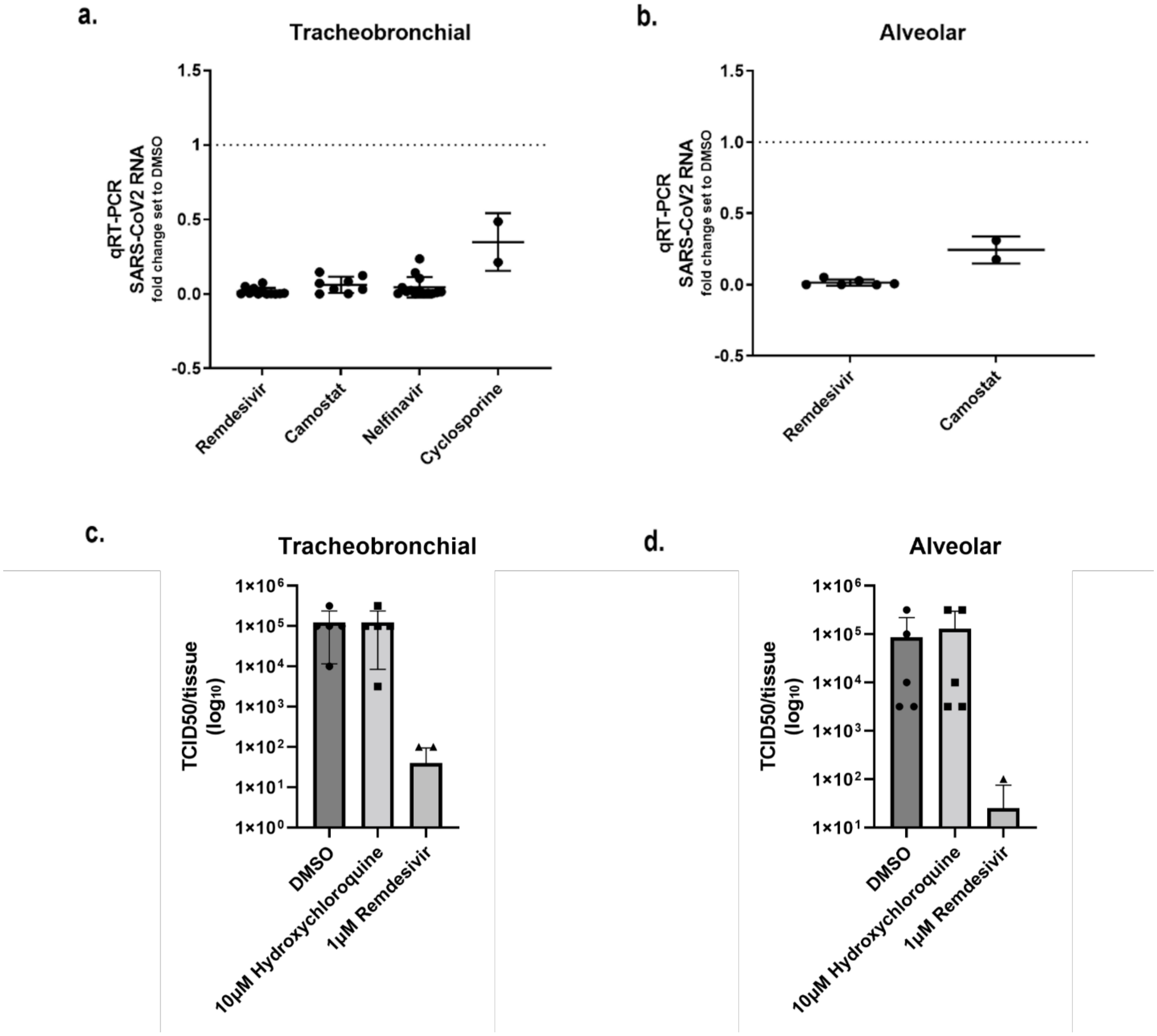

Supplemental Figure 10: Reduction in SARS-CoV-2 viral RNA or $TCID_{50}$ units in tracheobronchial or alveolar tissues treated with compounds. a,b) Intracellular SARS-CoV-2 viral RNA detection by qRT-PCR in **a)** tracheobronchial tissues or **b)** alveolar tissues treated with indicated compounds

at 10μM at 36 hpi. **c,d)** $TCID_{50}$ units measured from apical washes from **a)** tracheobronchial tissues or **b)** alveolar tissues treated with 10μM hydroxychloroquine or 1μM remdesivir at 48 hpi.

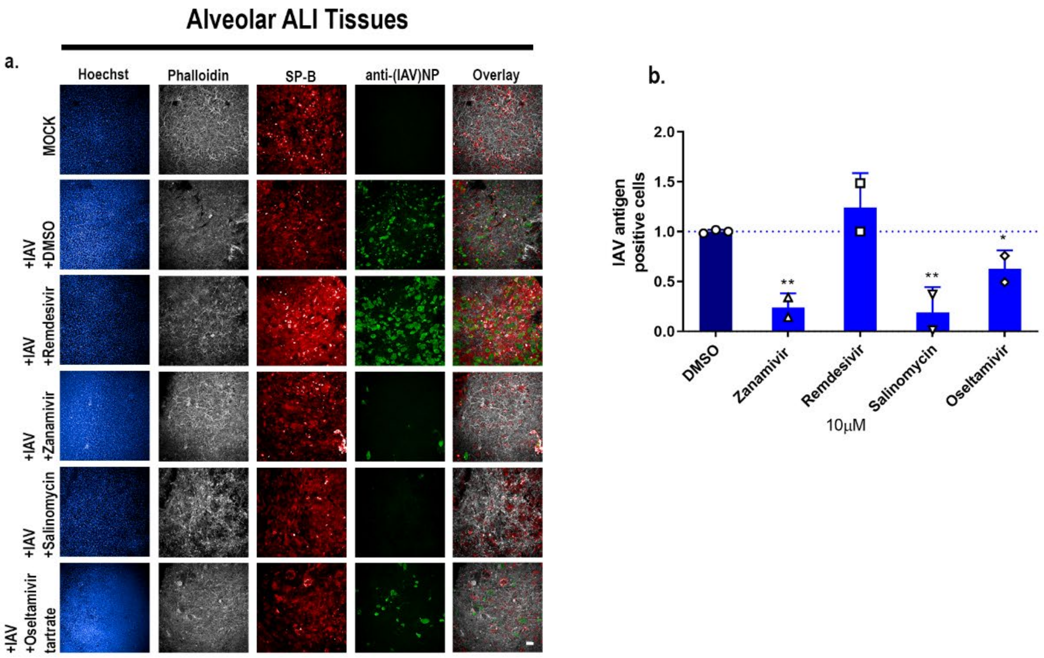

Supplemental Figure 11. Reduction in IAV viral antigen in alveolar tissues treated with different compounds. (a) IAV NP antigen staining in tissues treated with indicated compounds at 10μM at 24 hpi. Scale bar is 100 μm. **(b)** quantification of IAV NP positive cells from (a). Data are represented as M±SEM for a minimum of n=2 independent experiments and/or biological replicates; Student t-test of IAV or SARS-CoV-2 infected tissues vs. uninfected controls at each timepoint: *$p < 0.05$, **$p < 0.005$, ***$p < 0.0005$, ****$p < 0.00005$.

Supplemental Table 1:

**IFA/IHC Reagents**		
Antibody target	*Antibody type*	*Dilution*
α-tubulin (ciliated cell marker, rat mAb, ThermoFisher, MA1-80017)	Primary	1:200
SARS-CoV-2 N (rabbit mAb, Genetex, GTX635679)<br>SARS-CoV-2 S (rabbit mAb, Genetex, GTX135356)	Primary	1:100 (mixed 50/50 nucleocapsid + spike)
N 1C7C7 (SARS-CoV-2 marker, mouse mAb, Leinco, LT7000)	Primary	1:500
Surfactant protein B (ATII cell marker, rabbit pAb, Abcam ab40876)	Primary	1:100
AQP5+ (ATI cell marker, rabbit mAb, Abcam, ab92320)	Primary	1:100
MUC5B (Goblet cell marker, rabbit pAb, Sigma-Aldrich, HPA008246)	Primary	1:100
MUC5AC (Goblet cell marker, mouse mAb, Sigma-Aldrich, clone45M1, #M5293)	Primary	1:100
Cytokeratin 5 antibody (Basal cells marker, Conjugated, rabbit mAb, Abcam, ab193895, Alexa Fluor647)	Primary/ Secondary	1:100
Alexa Fluor 488 Phalloidin, ThermoFisher	Primary	1:40
Anti-Influenza A virus NP Mouse Monoclonal Antibody [clone: H16-L10-4R5 (HB-65), VWR]	Primary	1:1000
Hoechst 33342, ThermoFisher	N/A	10µg/ml
Goat anti-Mouse IgG (H+L), Goat anti-Rat IgG (H+L), Goat anti-Rabbit (H+L) Highly Cross-Adsorbed Secondary Antibody, Alexa Fluor 488, ThermoFisher	Secondary	1:300
Goat anti-Mouse IgG (H+L), Goat anti-Rat IgG (H+L), Goat anti-Rabbit (H+L) Highly Cross-Adsorbed Secondary Antibody, Alexa Fluor 567, ThermoFisher	Secondary	1:300
Goat anti-Mouse IgG (H+L), Goat anti-Rat IgG (H+L), Goat anti-Rabbit (H+L), Alexa Fluor 647, ThermoFisher	Secondary	1:300
DAPI, ThermoFisher, 62248	N/A	1:1000

Supplemental Table 2: Cell Identification Clusters

Tracheobronchial ALI Tissue: Cell Composition

Gene Markers	MKI67- ,TP63+, KRT5+	MKI67- ,TP63+, KRT5+, high EMT	MKI67- ,TP63+, KRT5+, KRT4+	MKI67- ,TP63+, KRT5+	MUC5B +, MUC5A C+, SCGB1 A1+,SP DEF+	EPCAM +, TP63- , BRIFA+, SCGB1 A1+	MKI67+	KRT5+/ TP63- /KRT13 +/KRT4 +	FOXJ1+ , TUBA1A +	KRT5+/ TP63- /KRT13 +/KRT4 +	EPCAM +, TP63- , BRIFA+, SCGB1 A1+
Cell-type Classification Cell Numbers Sequenced	Basal 1	Basal 2	Basal 3	Basal 4	Goblet	SupraB C-Club1	Cycling Basal	SupraB C1	Ciliated	SupraB C2	SupraB C-Club2
Cluster	0,6	7	1	2	3	4	5	8	9	10	11
Mock 48h-1 (Batch 1)	36,1420	535	1165	11	659	2	208	266	87	0	6
Mock 48h-2 (Batch 1)	25,1575	337	1074	16	585	2	229	272	80	0	6
Mock 72h-1 (Batch 2)	2868,18	320	1122	7	783	4	797	729	342	323	75
Mock 72h-1 (Batch 2)	2023,40	283	1112	10	425	12	578	22	230	190	35
Cell-type Classification Percentage of Total Cells	Basal 1	Basal 2	Basal 3	Basal 4	Goblet	SupraB C-Club1	Cycling Basal	SupraB C1	Ciliated	SupraB C2	SupraB C-Club2
Cluster	0,6	7	1	2	3	4	5	8	9	10	11
Mock 48h-1 (Batch 1)	33.129%	12.173%	26.507%	0.250%	14.994%	0.046%	4.733%	6.052%	1.980%	0.000%	0.137%
Mock 48h-2 (Batch 1)	38.086%	8.022%	25.565%	0.381%	13.925%	0.048%	5.451%	6.475%	1.904%	0.000%	0.143%
Mock 72h-1 (Batch 2)	39.063%	4.331%	15.187%	0.095%	10.598%	0.054%	10.788%	9.867%	4.629%	4.372%	1.015%
Mock 72h-1 (Batch 2)	41.593%	5.706%	22.419%	0.202%	8.569%	0.242%	11.653%	0.444%	4.637%	3.831%	0.706%
**Mean**	37.968%	7.558%	22.420%	0.232%	12.022%	0.097%	8.156%	5.709%	3.288%	2.051%	0.500%
**Standard Deviation**	3.07%	2.97%	4.44%	0.10%	2.57%	0.08%	3.09%	3.38%	1.35%	2.06%	0.38%

Alveolar ALI Tissue: Cell Composition

Gene markers	SLC34A2+, HOPX+, high NKX2-1, high AQP5, low PDPN	SLPI+, CAV1+, CAV2+, PDPN+ *5,7: +immune response genes*	SLPI+, CAV1+, CAV2+, PDPN+	SLC34A2+	HOPX+,PDPN + 11: + Cell Cycle Genes	PECAM1 + VWF+	DCN+ VIM+ PDGFRA+
Cell-type Classification Cell Numbers Sequenced	ATI, ATII	ATI 1	ATI 2	ATII 1	ATI intermediate	Endothelial	Fibroblast
Cluster #	0	1,5,7	8	3,10	4,11	6,9	2
Mock 48h-1 (Batch 1)	547	891,1,0	280	115, 59	203,9	116, 19	217
Mock 48h-2 (Batch 1)	385	506,0,0	218	207, 61	161,5	43, 16	166
Mock 72h-1 (Batch 2)	604	532,0,0	205	310, 19	539,18	302, 159	374
Cell-type Classification Percentage of Total Cells	ATI, ATII	ATI 1	ATI 2	ATII 1	ATI intermediate	Endothelial	Fibroblast
Cluster	0	1,5,7	8	3,10	4,11	6,9	2
Mock 48h-1 (Batch 1)	22.263%	36.304%	11.396%	7.082%	8.628%	5.495%	8.832%
Mock 48h-2 (Batch 1)	21.776%	28.620%	12.330%	15.158%	9.389%	3.337%	9.389%
Mock 72h-1 (Batch 2)	19.726%	17.374%	6.695%	10.745%	18.191%	15.056%	12.214%

**Mean**	21.255%	27.433%	10.140%	10.995%	12.069%	7.962%	10.145%	21.255%	27.433%	10.140%	10.995%
**Standard Deviation**	1.10%	7.77%	2.47%	3.30%	4.34%	5.09%	1.48%	1.10%	7.77%	2.47%	3.30%

Supplemental File 1: Differential and Shared Up/Down-regulated genes in SARS-CoV-2 or IAV infected ALI tissues.

Supplemental File 2: Top 10 Upregulated or Downregulated GSEA Pathways in SARS-CoV-2 or IAV infected ALI tissues.
