# Supplementary material for: Modeling SARS-CoV-2 and influenza infections and antiviral treatments in human lung epithelial tissue equivalents
Source: Commun Biol. 2022 Aug 12;5:810. doi: 10.1038/s42003-022-03753-7 (PMC9373898; doi:10.1038/s42003-022-03753-7)
Supplement: Supplementary file 2 — Description of Additional Supplementary Files [file 42003_2022_3753_MOESM2_ESM.pdf]

Description of Additional Supplementary Files

File Name: Supplementary Data 1

Description: Differential and Shared Up/Down-regulated genes in SARS-CoV-2 or IAV infected ALI tissues

File Name: Supplementary Data 2

Description: Top 10 Upregulated or Downregulated GSEA Pathways in SARS-CoV-2 or IAV infected ALI tissues.

File Name: Supplementary Data 3

Description: Raw data from Fig. 3, Fig. 4, Fig. 7, and Fig.8
