# Supplementary material for: Modeling SARS-CoV-2 and influenza infections and antiviral treatments in human lung epithelial tissue equivalents
Source: Commun Biol. 2022 Aug 12;5:810. doi: 10.1038/s42003-022-03753-7 (PMC9373898; doi:10.1038/s42003-022-03753-7)
Supplement: Supplementary file 6 — Reporting Summary [file 42003_2022_3753_MOESM6_ESM.pdf]

nature portfolio

Corresponding author(s): Emily Lee, Marc Ferrer

Last updated by author(s): May 30, 2022

Reporting Summary

Nature Portfolio wishes to improve the reproducibility of the work that we publish. This form provides structure for consistency and transparency in reporting. For further information on Nature Portfolio policies, see our Editorial Policies and the Editorial Policy Checklist.

Statistics

For all statistical analyses, confirm that the following items are present in the figure legend, table legend, main text, or Methods section.

n/a	Confirmed	
☐	☒	The exact sample size (n) for each experimental group/condition, given as a discrete number and unit of measurement
☐	☒	A statement on whether measurements were taken from distinct samples or whether the same sample was measured repeatedly
☐	☒	The statistical test(s) used AND whether they are one- or two-sided<br>*Only common tests should be described solely by name; describe more complex techniques in the Methods section.*
☐	☒	A description of all covariates tested
☐	☒	A description of any assumptions or corrections, such as tests of normality and adjustment for multiple comparisons
☐	☒	A full description of the statistical parameters including central tendency (e.g. means) or other basic estimates (e.g. regression coefficient) AND variation (e.g. standard deviation) or associated estimates of uncertainty (e.g. confidence intervals)
☐	☒	For null hypothesis testing, the test statistic (e.g. F, t, r) with confidence intervals, effect sizes, degrees of freedom and P value noted<br>*Give P values as exact values whenever suitable.*
☒	☐	For Bayesian analysis, information on the choice of priors and Markov chain Monte Carlo settings
☐	☒	For hierarchical and complex designs, identification of the appropriate level for tests and full reporting of outcomes
☒	☐	Estimates of effect sizes (e.g. Cohen's d, Pearson's r), indicating how they were calculated

Our web collection on statistics for biologists contains articles on many of the points above.

Software and code

Policy information about availability of computer code

Data collection	Illumina NexSeq550 Platform
Data analysis	The enrichment analysis was performed using R package EnrichR (libraries:BioPlanet_2019,KEGG_2021_Human,WikiPathways_2021_Human, MSigDB_Hallmark_2020, GO_Biological_Process_2021, GO_Molecular_Function_2021), fgsea (library c5.ontology gene sets all.v7.4) and clusterprofiler (library Go.db 2.1) packages with selected DEG list from the differential analysis (described above). The combined expression of the genes in each enrichment category was calculated using AddModuleScore function in Seurat packag. Differential Gene Expression (DEG) Analyses were applied by comparing 'tissue type', 'type of virus', 'Infection status' with Seurat FindAllMarkers, and FindMarkers functions. The visualization plots were generated using R packages (EnhancedVolcano and ggplot2). Illumina NexSeq550 Platform (Illumina). The raw data were demultiplexed and mapped to human reference genome with virus genomes using CellRanger (10x Genomics) with standard default pipeline parameters. Raw count matrix for each sample was imported into an R pipeline using Seurat v4 package. Low quality cells (< 200 genes, < 400 UMI, < 0.8 gene complexity (log10GenesPerUMI) and > 0.2 mitochondrial ratio) were filtered out from analysis. Additionally, genes which were expressed in less than 10 cells were excluded from downstream analysis. Doublet cells were further removed by running DoubletFinder R package. Data from each tissue were then normalized, scaled and log-transformed with Seurat packages using the SCTransform method.

For manuscripts utilizing custom algorithms or software that are central to the research but not yet described in published literature, software must be made available to editors and reviewers. We strongly encourage code deposition in a community repository (e.g. GitHub). See the Nature Portfolio guidelines for submitting code & software for further information.

Data

Policy information about availability of data

All manuscripts must include a data availability statement. This statement should provide the following information, where applicable:

- Accession codes, unique identifiers, or web links for publicly available datasets
- A description of any restrictions on data availability
- For clinical datasets or third party data, please ensure that the statement adheres to our policy

The datasets generated during and/or analysed during the current study are available from the corresponding authors on reasonable request.

Human research participants

Policy information about studies involving human research participants and Sex and Gender in Research.

Reporting on sex and gender	N/A
Population characteristics	N/A
Recruitment	N/A
Ethics oversight	N/A

Note that full information on the approval of the study protocol must also be provided in the manuscript.

Field-specific reporting

Please select the one below that is the best fit for your research. If you are not sure, read the appropriate sections before making your selection.

☒ Life sciences ☐ Behavioural & social sciences ☐ Ecological, evolutionary & environmental sciences

For a reference copy of the document with all sections, see nature.com/documents/nr-reporting-summary-flat.pdf

Life sciences study design

All studies must disclose on these points even when the disclosure is negative.

Sample size	N=3 for all main figures. scRNAseq was n=2 per replicate with several thousand cells sequenced per sample.
Data exclusions	Data excluded from virus exposed luminex profiling if sample did not have confirmed viral infection by either viral RNA or viral titer. (Except for mocks - those did not have viral infection)
Replication	Experiments throughout the paper were mostly conducted either at different dates by same group to confirm reproducibility or by multiple groups. e.g. SARS-CoV-2 infections were carried out by teams at TBRI, UPenn, and NCATS.
Randomization	N/A.
Blinding	Blinding was done via sample coding on luminex samples and compound studies were codes were assigned prior to sample processing and then data analyzed before decoding.

Reporting for specific materials, systems and methods

We require information from authors about some types of materials, experimental systems and methods used in many studies. Here, indicate whether each material, system or method listed is relevant to your study. If you are not sure if a list item applies to your research, read the appropriate section before selecting a response.

Materials & experimental systems

n/a	Involved in the study
☐	☒ Antibodies
☐	☒ Eukaryotic cell lines
☒	☐ Palaeontology and archaeology
☒	☐ Animals and other organisms
☒	☐ Clinical data
☒	☐ Dual use research of concern

Methods

n/a	Involved in the study
☒	☐ ChIP-seq
☒	☐ Flow cytometry
☒	☐ MRI-based neuroimaging

Antibodies

Antibodies used

α-tubulin (ciliated cell marker, rat mAb, ThermoFisher, MA1-80017)
SARS-CoV-2 N (rabbit mAb, Genetex, GTX635679)
SARS-CoV-2 S (rabbit mAb, Genetex, GTX135356)
N 1C7C7 (SARS-CoV-2 marker, mouse mAb, Leinco, LT7000)
Surfactant protein B (ATII cell marker, rabbit pAb, Abcam ab40876)
AQP5+ (ATI cell marker, rabbit mAb, Abcam, ab92320)
MUC5B (Goblet cell marker, rabbit pAb, Sigma-Aldrich, HPA008246)
MUC5AC (Goblet cell marker, mouse mAb, Sigma-Aldrich, clone45M1, #M5293)
Cytokeratin 5 antibody (Basal cells marker, Conjugated, rabbit mAb, Abcam, ab193895, Alexa Fluor647)
Alexa Fluor 488 Phalloidin, ThermoFisher
Anti-Influenza A virus NP Mouse Monoclonal Antibody [clone: H16-L10-4R5 (HB-65), VWR]
Hoechst 33342, ThermoFisher
Goat anti-Mouse IgG (H+L), Goat anti-Rat IgG (H+L), Goat anti-Rabbit (H+L) Highly Cross-Adsorbed Secondary Antibody, Alexa Fluor 488, ThermoFisher
Goat anti-Mouse IgG (H+L), Goat anti-Rat IgG (H+L), Goat anti-Rabbit (H+L) Highly Cross-Adsorbed Secondary Antibody, Alexa Fluor 567, ThermoFisher
Goat anti-Mouse IgG (H+L), Goat anti-Rat IgG (H+L), Goat anti-Rabbit (H+L), Alexa Fluor 647, ThermoFisher
DAPI, ThermoFisher, 62248

Validation

Antibodies were validated in-house prior to use by testing in different tissue types for specificity. Antibodies that showed reactivity in inappropriate tissue types were not used in the study.

Eukaryotic cell lines

Policy information about cell lines and Sex and Gender in Research

Cell line source(s)

Vero E6 cells were obtained from the American Type Culture Collection (ATCC CRL-1586), Rhesus monkey kidney epithelial cells LLC-MMK2, overexpressing SIAT1 were obtained from Dr. Jonathan Yewdell at NIAID. Vero-TMPRSS2 cells were purchased from BPS Bioscience. Primary human ALI lung tissues were purchased from MatTek.

Authentication

None of the immortalized cell lines used were further authenticated at NCATS. Primary human ALI tissues were validated via immunostaining and scRNAseq when applicable.

Mycoplasma contamination

Primary ALI lung tissues were screened for mycoplasma before receipt for use. Immortalized cell lines were not tested for mycoplasma contamination.

Commonly misidentified lines
(See ICLAC register)

N/A.
